# Alkaloid-Based Isoxazolylureas: Synthesis and Effect in Combination with Anticancer Drugs on C6 Rat Glioma Model Cells

**DOI:** 10.3390/molecules29143246

**Published:** 2024-07-09

**Authors:** Gulim K. Mukusheva, Roza I. Jalmakhanbetova, Altynay Zh. Shaibek, Manshuk S. Nurmaganbetova, Aigerym R. Zhasymbekova, Oralgazy A. Nurkenov, Ekaterina A. Akishina, Irina A. Kolesnik, Evgenij A. Dikusar, Tatiana I. Terpinskaya, Vladimir A. Kulchitsky, Vladimir I. Potkin, Alexander L. Pushkarchuk, Dmitry A. Lyakhov, Dominik L. Michels

**Affiliations:** 1 Faculty of Chemistry, Karaganda Buketov University, Karaganda 100024, Kazakhstan; mukusheva1977@list.ru (G.K.M.); altu_ekosya@mail.ru (A.Z.S.); manshuk_nur@mail.ru (M.S.N.); aigera-93-93@mail.ru (A.R.Z.); 2 Faculty of Natural Sciences, L.N. Gumilyov Eurasian National University, Astana 010000, Kazakhstan; rozadichem@mail.ru; 3 Institute of Organic Synthesis and Coal Chemistry of the Republic of Kazakhstan, Karaganda 100008, Kazakhstan; nurkenov_oral@mail.ru; 4 Institute of Physical Organic Chemistry, National Academy of Sciences of Belarus, 220072 Minsk, Belarus; irynakolesnik93@gmail.com (I.A.K.); evgen_58@mail.ru (E.A.D.); potkin@ifoch.bas-net.by (V.I.P.); alexp51@bk.ru (A.L.P.); 5 Institute of Physiology, National Academy of Sciences of Belarus, 220072 Minsk, Belarus; terpinskayat@mail.ru (T.I.T.); kulchitski48@mail.ru (V.A.K.); 6 Computer, Electrical and Mathematical Science and Engineering Division, King Abdullah University of Science and Technology, Thuwal 23955-6900, Saudi Arabia; dmitry.lyakhov@kaust.edu.sa (D.A.L.); dominik.michels@kaust.edu.sa (D.L.M.)

**Keywords:** anabasine, cytisine, ureas, isoxazole, cytotoxic activity, antiproliferative activity, drug synergism, quantum chemical modeling, molecular docking

## Abstract

Alkaloid-based urea derivatives were produced with high yield through the reaction of anabasine and cytisine with isoxazolylphenylcarbamates in boiling benzene. Their antitumor activity, in combination with the commonly used five anticancer drugs, namely cyclophosphane, fluorouracil, etoposide, cisplatin, ribomustine with different mechanisms of action, was investigated. Based on the quantum chemical calculations data and molecular docking, hypotheses have been put forward to explain their mutual influence when affecting C6 rat glioma model cells.

## 1. Introduction

Cancer is one of the leading causes of death worldwide. Chemotherapy has been widely used as the primary therapy, particularly against inoperable cancer or as an adjunct therapy before or after another treatment. However, it is usually accompanied by negative side effects. The combination of classical anticancer drugs with synergistic adjuvants makes it possible to reduce the therapeutic dose and thereby reduce the toxicity of chemotherapy. Therefore, the approach of using synergistic adjuvants is being seriously considered. This strategy is based on the susceptibility of different molecular pathways involved in the genesis of a particular disease to the different mechanism of action of each individual drug to improve treatment efficacy and reduce the development of drug resistance [1,2].

The main goal when developing a drug combination is to achieve an effect significantly greater than the additive effect of the individual drugs, thereby reducing dosage and toxicity. On the other hand, many types of complementary medicines may be ineffective. Worse, they can produce unintended adverse side effects or alter the actions of conventional medical treatments by inhibiting or amplifying their effects to dangerous levels [3,4]. Therefore, the search for new effective chemotherapeutic drug combinations, and the study of the mechanisms underlying their mutually influencing processes, seem to be an urgent task of medicinal chemistry.

The isoxazole ring is often used as a central core of compounds with anticancer effects acting as inhibitors of numerous targets including aromatase, tyrosine kinase, thymidylate, ERα, PLA2, HDAC, HER2, HSP90 inhibition and apoptosis induction [5,6,7]. The structural features of isoxazole make it possible for multiple non-covalent interactions, especially hydrogen bonds (hydrogen bond acceptor N and O), π-π stacking (unsaturated five-membered ring) and hydrophilic interactions [8]. The authors of this article have been conducting research on molecular design of structures, synthesis and biotesting of new synergistic adjuvants in compositions with first-line anticancer drugs for a number of years. As a result, it was found that some isoxazole derivatives are promising candidates for a synergist’s role [9,10,11]. Previously conducted quantum chemical calculations of structural and electronic changes occurring in the cisplatin–adjuvant system based on morpholinium and 4-methylpiperazinium 4,5-dichloroisothiazol-3-carboxylates allowed us to assume that the synergistic effect appeared due to the conjugation of cisplatin with adjuvant through the relocation of frontier molecular orbitals, as well as an increase in the conjugate’s dipole moment, which led to a change in the interaction character with the cellular target and an increase in the bioactivity of the system [12].

Continuing our research in this area, we expanded the scope of such studies to include other chemotherapy drugs that are used in modern oncology, as well as new promising 1,2-azole derivatives possessing synergistic effects.

## 2. Results and Discussion

### 2.1. Chemistry

**Scheme 1:**
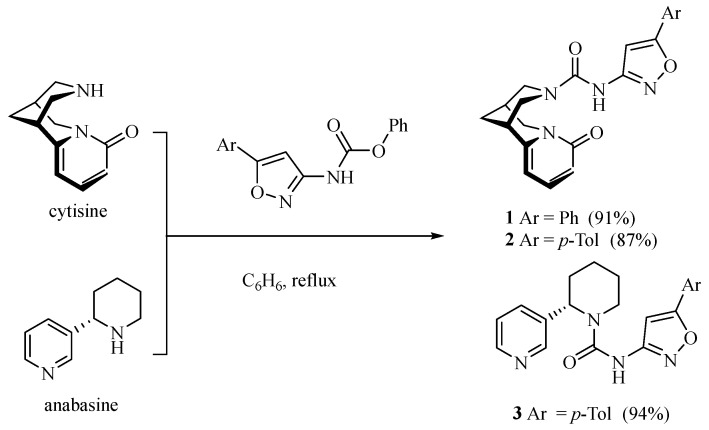
Synthesis of anabasine and cytisine ureas with isoxazole fragments.

We previously discovered that some azolylcarbamides and carbamates can exhibit a synergistic effect in compositions with antitumor substances [13]. In this work, to obtain 5-arylisoxazolyl ureas with alkaloid fragments, an approach we used in [11], was chosen. (Scheme 1). The starting 5-phenylisoxazolylcarbamate was synthesized through the reaction of 5-phenylisoxazole-3-carbonyl azide with phenol, as described by us before [11]. Isoxazolylphenylcarbamates were further introduced in the reaction with cytisine and anabasine to obtain **1-3** in 87–94% yields. NMR, MS, IR spectra of synthesized ureas are available in the Supplementary Materials.

### 2.2. Evaluation of Antitumor Activity

Determination of anticancer activity of isoxazolylureas **1-3** drugs and their binary mixtures on rat glioma cell line was carried out using flow cytometry analysis. C6 cell line is considered to be a safe and popular glioma model in the literature, providing a good simulation of glioblastoma multiforme [14].

**Figure 1:**
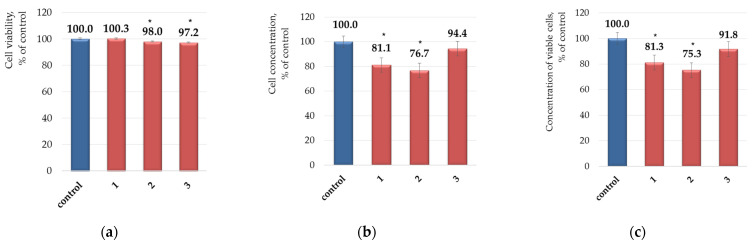
Effect of **1-3** (200 µM) on the viability (**a**), concentration (**b**) and concentration of viable cells (**c**) of C6 glioma, * *p* < 0.05.

At the beginning, the antitumor effect of synthesized isoxazolylureas was studied. The compounds **1-3** are found to have a little effect on the viability of C6 glioma cells (Figure 1a). Suppression of cell proliferation was observed: the drugs reduced the number of cells in the samples by 19, 23 and 6% compared to the control (Figure 1b). The total number of live cells in the samples decreased by 19, 25 and 8%, respectively (Figure 1c). Thus, the anticancer effect of isoxazolylureas **1-3** is determined mainly by the antiproliferative action of the compounds. Alkaloids anabasine and cytisine in doses of 100–500 μM had a weak inhibitory effect on cells or have no effect.

Hence, even at such a fairly high dose, compounds **1-3** exhibited weak cytotoxicity along with an antiproliferative effect on tumor cells. This makes them suitable candidates for the role of low-toxic synergists of anticancer drugs.

**Figure 2:**
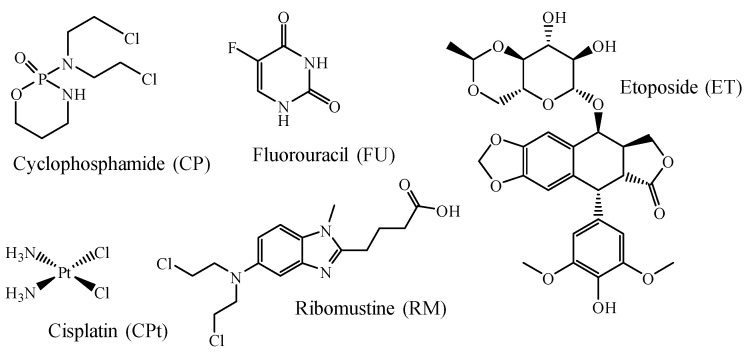
Anticancer drugs used in composition with isoxazolylurea adjuvants.

A study of the synergistic effects of the five commonly used anticancer drugs, namely cyclophosphamide (**CP**), fluorouracil (**FU**), etoposide (**ET**), cisplatin (**CPt**), ribomustine (**RM**), with different mechanisms of action, was undertaken (Figure 2).

The antitumor activity of combinations at fixed drug and isoxazolylurea concentrations was assessed to identify the most promising binary mixture. We used drugs and isoxazolylureas at concentrations of 5 and 200 μM, respectively, and compared their combined effect to a tenfold increased drug dose (50 μM).

**Figure 3:**
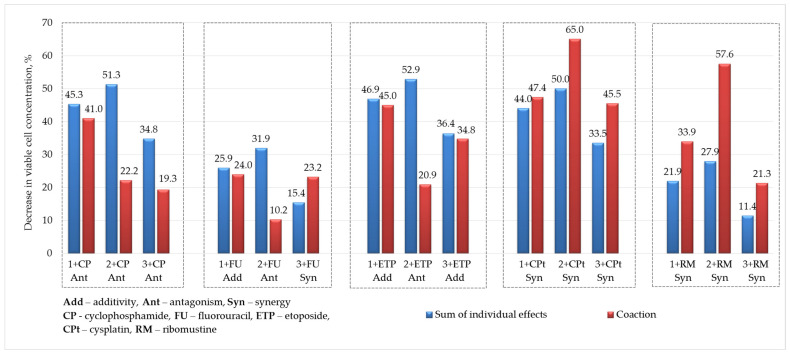
Decrease in viable C6 rat glioma cell concentration for binary mixtures and sum of the effects of an individual compound and drug (C_1-3_ = 200 µM, C_drug_ = 5 µM; number of repetitions for drug–urea composition in each experiment n = 8). Additivity—the effect of drugs combination equals the sum of their effects when given alone; antagonism—drug combinations show adverse results of a decrease in antitumor activity compared to the sum of individual effects; synergy—agents given together produce higher antitumor activity than the sum of individual effects.

The results of the study are summarized in the Table 1 and Figure 3. As can be seen from the experiment, compound **1** in combination with all drugs except ribomustine showed the effect of additive synergy, demonstrating an activity level approximately equal to that of the sum of each compound separately. Compositions with isoxazolylurea **2** showed a clear example of antagonism in combination with the drugs cyclophosmamide, fluorouracil and etoposide, inhibiting the pharmacological activity of each other or at least one of the compounds, and resulting in 2–3 fold decrease of antiprofilerative action compared to the sum of the effects of the two compounds. At the same time, the combination of compound **2** with cisplatin and ribomustine led to the opposite result: the synergistic effect was realized in a significant increase of the drug’s action. The cytostatic activity of the binary mixture **1+RM** (5 μM) exceeded the effect of the drug at a 10-fold increased dose (50 μM) by 22%. Generally, the most pronounced synergistic effect was observed in the case of ribomustine mixtures with all synthesized compounds.

The anabasine derivative **3** showed a synergistic effect with fluorouracil, cisplatin and ribomustin increasing the effectiveness of the drug by 8–12% and distinct antagonism with cyclophosphamide reducing the drug’s activity by 15%. In the combination with etoposide, additive action is noted.

Generally, a synergistic effect is observed to varying degrees for all ureas **1-3** when using binary mixtures with alkylating chemotherapy drugs cisplatin and ribomustine. Antagonism and the tendency to antagonism are noted for the remaining drugs, except for the combination of isoxazolylurea **3** with fluorouracil.

Antagonism is known to occur through various means. The most common type is receptor antagonism, where one drug acts as an antagonist to block the effects of another drug by binding to the same receptor. This prevents the agonist drug from activating the receptor and producing its intended effect. Some drugs follow chemical antagonism, which involves the coupling of two drugs to form an inactive product, etc. [15].

To interpret the observed experimental picture, quantum chemical modeling of binary drug–urea mixtures and molecular docking of isoxazolylureas was carried out, taking into account targets that determine the mechanism of drugs action.

### 2.3. Quantum Chemical Modeling

Based on the assumption that the effect of the combined action of drugs can be mediated by the formation of non-covalent drug–adjuvant conjugates, as was shown before [12], we determined structural and electronic changes that occur in the system of two conjugated molecules. DFT/B3LYP-D3/cc-pvdz/LanL2DZ(Pt) level of theory was used for the calculation of molecules’ optimal geometry, dipole moment, localization and energy characteristics of frontier molecular orbitals (FMO). Calculations were carried out both for individual compounds and their conjugates, with consideration given to aqueous medium, which simulates situation in living cells.

**Figure 4:**
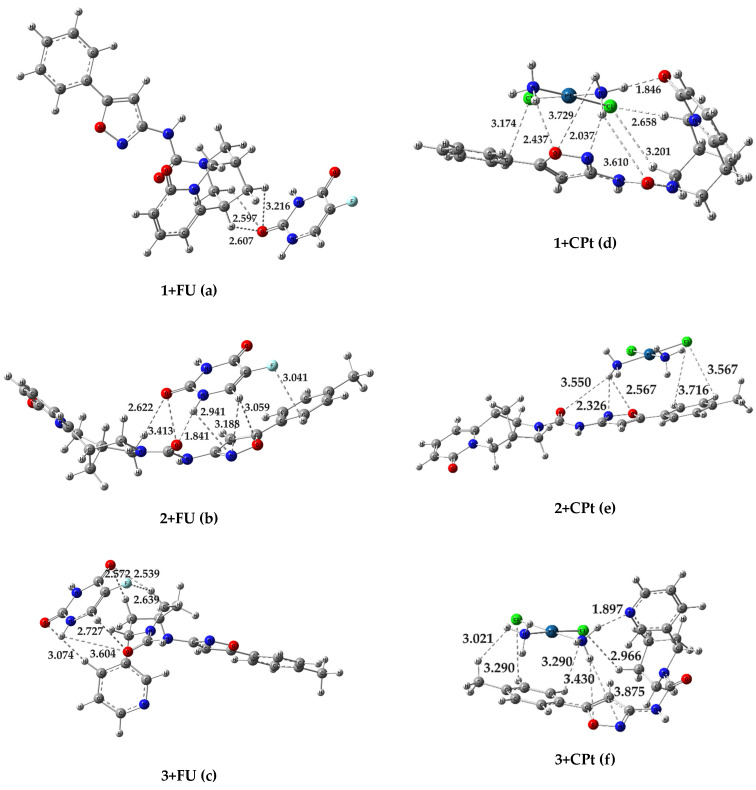
Calculated optimized structures with consideration given to aqueous medium for conjugates **1-3** with fluorouracil and cysplatine.

The optimized geometry of the drug–urea conjugates is presented in Figure 4, and energy characteristics in Table 1 and Table 2. The energy characteristics of conjugates formed from the initial components were calculated using formula (Table 2): ∆E_f_ = Σ[E_f products_] − Σ[E_f reactants_].

It follows from calculation data that drug molecules (**FU** and **CPt**) and urea derivatives **1-3** form conjugates with shortened interatomic distances due to non-covalent interactions caused by hydrogen and van der Waals bonds (Figure 4a–f). As can be seen from Figure 4, the structure of isoxazolylurea **2** differs from the other two in its most linear spatial arrangement, which results in the varying degrees of conjugates’ stability due to the participation of different atoms in intermolecular bond formation. Thus, in the case of fluorouracil, the most stable complex is observed with compound **2** with ∆E_f_ = −16.88 kcal/mol (Table 2). Hydrogen bonds are expressed between the oxygen and nitrogen atoms of the urea fragment, and between the nitrogen atom of isoxazole heterocycle and the N-H and C=O fragments of fluorouracil C=O_urea_···H-N/N_isox_···H-N/C=O···H-N_urea_ with interatomic distances of 1.84, 2.94 and 3.41 Å, respectively. Other non-covalent interactions represent weak unconventional C-H···O, C-H···N, C-H···F hydrogen bonds with interatomic distances of 2.62–3.19 Å. The formation of conjugates **1+FU (a)** and **3+FU (c)** does not involve atoms of the isoxazole heterocycle; only weak, unconventional hydrogen bonds with atoms of alkaloid fragments are generated (2.53–3.60 Å). As for conjugates with cisplatin, the opposite situation is observed: **1+CPt (d)** and **3+CPt (f)** turned out to be more stable with ∆E_f_ = −24.96 and −21.18 kcal/mol (Table 2). In contrast to conjugate **2+CPt (e),** the formation of hydrogen bonds involved the nitrogen atom of the pyridine heterocycle of anabasine and the oxygen atom of the cytisine C=O group, in addition to the N, O atoms of the isoxazole heterocycle and the urea fragment. The above allows us to conclude that the formation of stable complexes leads to the manifestation of antagonistic effects in the drug–urea system.

The energies of frontier molecular orbitals (FMO) are often considered as important descriptors for determining the biological activity of the molecule. One of the key characteristics in FMO theory is the difference between the energies of HOMO and LUMO (energy gap ΔE). The obtained values calculated using DFT/B3LYP-D3/cc-pvdz/LanL2DZ(Pt) level of theory are given in Table 2. Generally, the formation of all conjugates is accompanied by a significant decrease in ∆E: when going from fluorouracil FU to conjugates, the average value of decrease is 0.88 eV, from cisplatine to its conjugates—0.37 eV. This indicates an increase in reactivity of the system, which could mean more active binding with biological sites. It is worth mentioning that the decrease of ΔE for cisplatin conjugates occurs due to an increase in the HOMO energy by 0.47 eV, which contributes to the realization of their nucleophilic potential.

Global reactivity descriptors such as electronegativity (χ), chemical potential (μ), chemical softness–hardness (η and S) and electrophilic index (ω) are highly successful in predicting stability properties and reactivity trends of the molecular systems. The formal definitions of all these descriptors and working equations for their computation have been described in [16]. From theoretical calculations, it was found that chemical hardness (softness) value decreases (increases) when moving from drugs to conjugates, which indicates an increase in their chemical reactivity. The values of ω for conjugates shows that the electrophilicity of binary systems with fluorouracil increases, while with cisplatin, on the contrary, it decreases (Table 3).

The work in [12] provides data on the correlation between an increase in the potentiating activity of cisplatin complexes with heterocyclic compounds (having a rigid conformational structure) and an increase in the values of their calculated dipole moments, in comparison to pure cisplatin. In this work, acylurea derivatives have the possibility of conformational rotation of C-C, C-N and N-N bonds, and no such correlation is observed.

**Figure 5:**
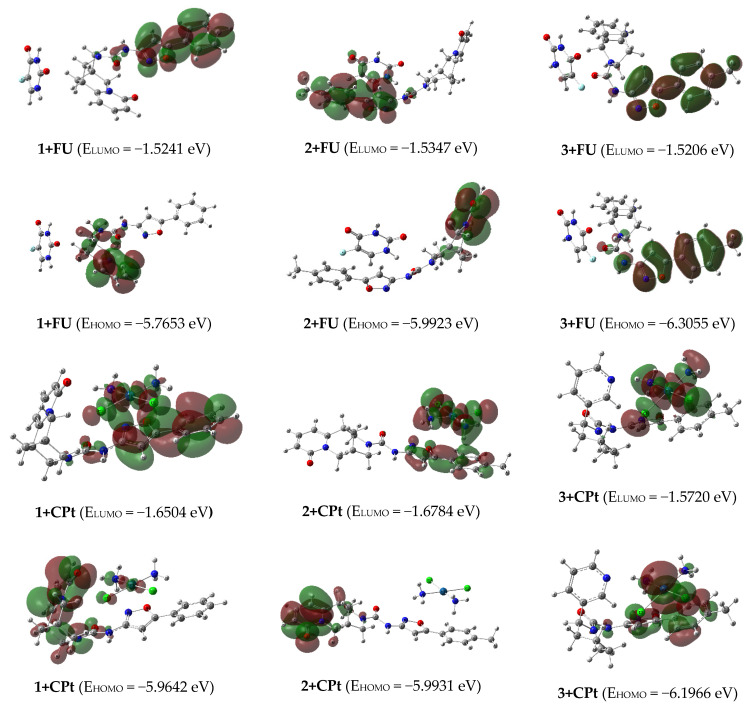
Localizations of HOMO and LUMO in molecules of conjugates in the form of 3D isosurfaces, with consideration given to water.

Determining the localization of FMO is important for establishing preferred directions and regions of the molecule for attack by nucleophiles and electrophiles. Results of calculating the localization of HOMO and LUMO are shown in the form of 3D isosurfaces (Figure 5).

The formation of conjugates with fluorouracil **1+FU**, **2+FU**, **3+FU** leads to the displacement of LUMO from the drug molecule and its localization on isoxazole heterocycles (Figure 5). Since LUMO determines interaction with target nucleophilic sites, this type of binding will be realized through the isoxazole heterocycle. Binding to electrophilic protein sites determines the localization of HOMO in conjugate. Calculations for conjugate molecules showed that HOMO in all fluorouracil conjugates is completely localized on isoxazole heterocycle or cytisine fragments, but not on the drug molecule. This means that the first act of interaction with the electrophilic and nucleophilic regions of protein sites is realized mainly due to the adjuvant molecule. In addition, based on previous findings, the **2+FU** complex turned out to be the most stable, which likely interferes with the drug molecule’s own action and makes the resulting conjugate the least effective against biological targets.

As for cisplatin conjugates **1+CPt** and **2+CPt,** LUMO are uniformly distributed between the isoxazole heterocycle and the cisplatin molecule, and HOMO are located on the cytisine fragment. Meanwhile the cisplatin molecule is responsible for interaction with nucleophilic sites in the **3-CPt** conjugate, and LUMO are concentrated here on the drug molecule. In contrast to **1+CPt** and **2+CPt** conjugates, HOMO in **3-CPt** do not affect the alkaloid fragment, being dispersed between cisplatin and the isoxazole heterocycle. All the given facts may indicate a coordinated mechanism of action of cisplatin and the adjuvant in binary mixtures towards biological targets.

### 2.4. Docking Studies

Thymidylate synthase is one of the key enzymes in carcinogenesis and, for this reason, is a critical target for cancer chemotherapy. As previously noted, the main mechanism of action of fluorouracil is its conversion into 5-fluoro-2-deoxy-5-monophosphate (FdUMP), resulting in the inhibition of TS and DNA synthesis. In order to study the possibility of thymidylate synthase inhibition by isoxazolyl ureas **1-3** as a process competing with the binding of fluorouracil and its metabolite to the same target, molecular docking was carried out using software packages AutoDock/Vina 4.2.6 and CHIMERA 1.16, Biovia Discovery Studio 2024 for the visualization of the docked pose. The crystal structure of target protein TS (mouse thymidylate synthase, PDB ID: 5FCT) was downloaded from the Protein Data Bank.

The efficiency of interactions was evaluated based on the changes observed in predicted binding energies and bonds formed with the active site residues of TS.

**Figure 6:**
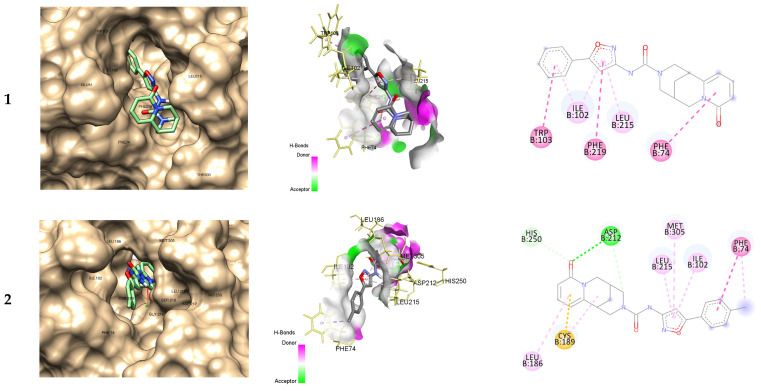

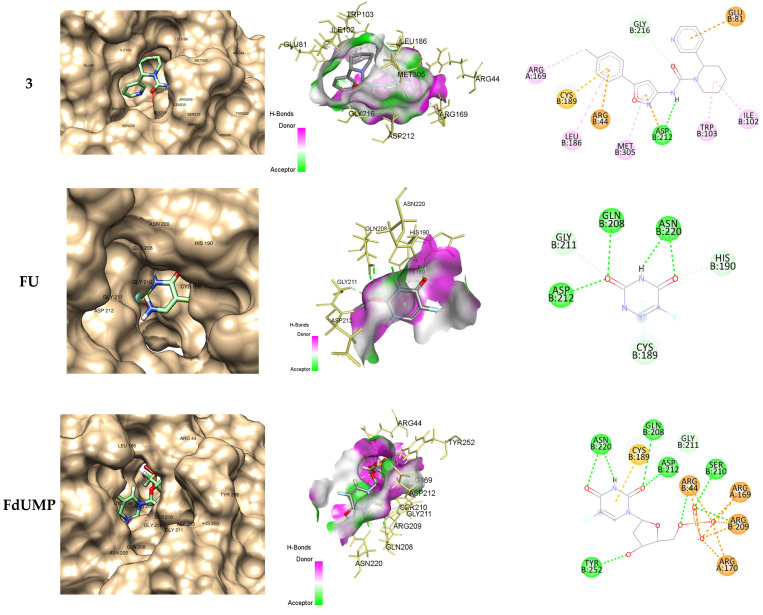
Binding interactions of isoxazolyl ureas **1-3** and fluorouracil **FU**, and its metabolite 5-fluoro-2′-deoxyuridine-5′-monophosphate **FdUMP** with thymidylate synthase.

As can be seen from Figure 6 and Table 4, all isoxazolyl ureas demonstrate a good binding degree to the thymidylate synthase protein site, forming an extensive system of residue interactions. The binding energy of **1-3** with tymidylate synthase is −9.3, −9.9, −10.0 kcal/mol, respectively, which even exceeds that of fluorouracil (−5.1 kcal/mol). Generally, ureas **1-3** form stable complexes with thymidylate synthase protein, mainly due to the hydrophobic interactions of electron-rich π systems of isoxazole heterocycle and aromatic rings with aminoacid residues, rather than hydrogen bonds, as occurs with fluorouracil and its metabolite.

It was observed that ureas **2**, **3**, as well as **5-FU** and **FdUMP**, occupied similar protein site-forming bonds with ASN212, CYS189, ARG169 and ARG44 amino acid residues (Table 4), which confirms the possibility of competing binding processes with the thymidylate synthase site. Meanwhile, for urea **1**, the weakest binding was observed and it also interacts with other amino acid residues of the target protein.

Considering the high binding energy with TS of all ureas, molecular docking data cannot explain such a different manifestation of fluorouracil in the composition with adjuvants (both antagonism and synergism are observed). Ureas can probably be multitarget ligands that attack other proteins as well. In addition, summing up the data on energy characteristics of the fluorouracil–urea conjugates formation and docking data on binding energy with TS, we can assume that adjuvants will instead bind the FU into a stable complex, preventing the drug from exerting its effect (ΔE_f_ ranges from −6.19 to −16.88 kcal/mol); this especially applies to isoxazolylurea **2**.

## 3. Materials and Methods

### 3.1. General Chemistry Section

UV spectra were recorded on a Varian Cary 300 spectrophotometer using quartz cuvettes with *l* = 1 cm. The concentration of the studied compounds in methanol was 4 × 10^−5^–1 × 10^−4^ M. IR spectra were registered on a Thermo Nicolet Protege 460 FT-IR spectrometer (Nicolet, Waltham, MA, USA) in KBr pellets.

^1^H and ^13^C NMR spectra were acquired on a Bruker Avance 500 spectrometer (Bruker, Bremen, Germany) in CDCl_3_. The residual solvent signals (CDCl_3_, δH 7.26, δC 77.2 ppm) were used as an internal standard. The assignment of signals in the ^13^C NMR spectra was performed using the DEPT technique.

Liquid chromatography–mass spectrometry spectra were recorded on an Agilent 1200 LC-MS system with an Agilent 6410 Triple Quad Mass Selective Detector (Agilent Technologies Inc., Santa Clara, CA, USA) with electrospray ionization in the positive ion registration mode (MS2 scanning mode). An Agilent ZORBAX Eclipse XDB-C18(4.6 × 50 mm, 1.8 μm) column (Agilent Technologies Inc., USA) was used. The mobile phase was MeCN–H_2_O + 0.05% HCO_2_H, with gradient elution from 40 to 90% MeCN in 10 min. A flow rate of 0.5 mL/min was used.

Elemental analysis was performed on a Vario MICRO cube CHNS-analyzer (Elementar, Langenselbold, Germany). Melting points were determined on a Kofler bench (VEB Wägetechnik Rapido PHMK, Radebeul, Germany).

The optical activity of the compounds was measured on a polarimeter MCP 100 (Anton Paar, Graz, Austria).

Reagents and solvents used were of analytical grade, with the content of the main component being more than 99.5%. Phenyl (5-arylisoxazol-3-yl)carbamate was synthesized according to the previously described procedures [11].

(–)-Anabasine (colorless viscous liquid, turning yellow in air and in light; bp 276 °C at 760 mmHg, 104–105 °C at 2 mmHg; d_20_ 1.0455, n_D_ 1.5430, [α]_D_^20^–82°) was isolated from anabasine hydrochloride (commercial product of Shymkentbiopharm, Kazakhstan) as an individual isomer.

(–)-Cytisine (commercial product of Shymkentbiopharm, Kazakhstan) is a white crystalline substance that crystallizes from acetone in rhombic prisms, mp. 153 °C, [α]_D_^20^ = −119° (in aqueous solution).

#### General Procedure for the Synthesis of Anabasine and Cytisine Ureas **1-3**

A mixture containing 1.6 g of anabasine (or 1.9 g of cytisine, 10 mmol) and 10 mmol of phenyl (5-arylisoxazol-3-yl)carbamate in 30 mL of benzene was boiled and stirred for 6 h. The solvent was removed in vacuum. Diethyl ether (50 mL) was added to the oily residue, and the mixture was boiled until complete crystallization had occurred. The crystalline product was separated by filtration, washed with ether and dried in air at room temperature.

(1R,5S)-3-(5-phenylisoxazole-3-carbonyl)-1,2,3,4,5,6-hexahydro-8H-1,5-methanopyrido [1,2-a][1,5]diazocin-8-one (**1**): beige solid; yield 91%; mp 241–242 °C; [α]_D_^25^ −193°; UV (MeOH c = 7·10^−5^ M) λ_max_ (log ε): 243 (4.26), 260 (4.38), 311 (3.87); IR (KBr) ν 3313, 3180, 2933, 2867, 1692, 1653, 1623, 1595, 1576, 1539, 1424, 1341, 1265, 1222, 1187, 1102, 943, 810, 797, 770, 687 cm^−1^; ^1^H NMR (CDCl_3_, 500 MHz) *δ* 1.97–2.04 (2H, m, 13-H_2_), 2.56 (1H, bs, 5-H), 3.10 (1H, bs, 1-H), 3.19–3.25 (2H, m), 3.90 (1H, dd, *J* = 15.6, 6.6 Hz, 6-H), 4.30–4.35 (2H, m), 4.46 (1H, d, *J* = 13.00 Hz), 6.02 (1H, d, *J* = 6.8 Hz, 11-H), 6.45 (1H, dd, *J* = 9.0, 1.0 Hz, 9-H), 7.07 (1H, s, 4′-H_isox_), 7.21 (1H, dd, *J* = 9.0, 6.9 Hz, 10-H), 7.40–7.45 (3H, m, 3,4,5-CH_Ar_), 7.70 (2H, dd, *J* = 8.0, 1.5 Hz, 2,6-CH_Ar_), 9.25 (1H, s, C(O)NH); ^13^C NMR (CDCl_3_, 125 MHz) *δ* 26.00 (12-CH_2_), 27.57 (5-CH), 34.73 (1-CH), 49.05 (CH_2_), 50.54 (CH_2_), 51.36 (CH_2_), 95.10 (4′-CH_isox_), 105.73 (11-CH), 117.66 (9-CH), 125.71 (2,6-CH_Ar_), 129.12 (3,5-CH_Ar_), 130.39 (1CH_Ar_), 139.03 (10-CH), 127.58, 148.72, 154.35, 160.55, 163.62, 169.12 (6C_quater_); MS *m*/*z* (*I*_rel_, %) 377.20 [M+H]^+^ (100); Anal. calcd. for C_21_H_20_N_4_O_3_ (376.41): C, 67.01; H, 5.36; N, 14.88%. Found: C, 67.22; H, 5.47; N, 14.61%.

(1R,5S)-3-(5-(p-tolyl)isoxazole-3-carbonyl)-1,2,3,4,5,6-hexahydro-8H-1,5-methanopyrido [1,2-a][1,5]diazocin-8-one (**2**): beige solid; yield 87%; mp 255–257 °C; [α]_D_^25^ −250°; UV (MeOH c = 7·10^−5^ M) λ_max_ (log ε): 240 (4.20), 266 (4.43), 310 (3.87); IR (KBr) ν 3146, 3029, 2925, 2886, 2776, 1650, 1625, 1611, 1564, 1545, 1507, 1428, 1337, 1257, 1229, 1143, 1061, 789 cm^−1^; ^1^H NMR (CDCl_3_, 500 MHz) *δ* 1.96–2.04 (2H, m, 13-H_2_), 2.36 (3H, s, CH_3_), 2.55 (1H, bs, 5-H), 3.09 (1H, bs, 1-H), 3.21 (1H, d, *J* = 12.6 Hz, CH_2_), 3.23 (1H, dd, *J* = 12.9, 1.9 Hz, CH_2_), 3.90 (1H, dd, *J* = 15.6, 6.6 Hz, 6-H), 4.28–4.35 (2H, m, CH_2_), 4.45 (1H, d, *J* = 13.00 Hz, CH_2_), 6.01 (1H, dd, *J* = 6.9, 1.0 Hz, 11-H), 6.44 (1H, dd, *J* = 9.0, 1.2 Hz, 9-H), 7.02 (1H, s, 4′-H_isox_), 7.20 (1H, dd, *J* = 9.0, 6.8 Hz, 10-H), 7.22 (2H, d, *J* = 8.0 Hz, 3,5-CH_Ar_), 7.59 (2H, d, *J* = 8.1 Hz, 2,6-CH_Ar_), 9.27 (1H, s, C(O)NH); ^13^C NMR (CDC l_3_, 125 MHz) *δ* 21.58 (CH_3_), 25.98 (12-CH_2_), 27.55 (5-CH), 34.72 (1-CH), 49.06 (CH_2_), 50.45 (CH_2_), 51.33 (CH_2_), 94.50 (4′-CH_isox_), 105.69 (11-CH), 117.64 (9-CH), 125.64 (2,6-CH_Ar_), 129.80 (3,5-CH_Ar_), 139.00 (10-CH), 124.87, 140.70, 148.71, 154.32, 160.53, 163.60, 169.31 (7C_quater_); MS *m*/*z* (*I*_rel_, %) 391.20 [M+H]^+^ (100); Anal. calcd. for C_22_H_22_N_4_O_3_ (390.44): C, 67.68; H, 5.68; N, 14.35%; Found: C, 67.91; H, 5.75; N, 14.09%.

(S)-2-(pyridin-3-yl)-N-(5-(p-tolyl)isoxazol-3-yl)piperidine-1-carboxamide (**3**): beige solid; yield 94%; mp 114–116 °C; [α]_D_^25^ −139°; UV (MeOH c = 8·10^−5^ M) λ_max_ (log ε) 264 (4.43); IR (KBr) ν 3254, 3216, 3097, 3063, 2946, 2861, 1671, 1628, 1571, 1555, 1426, 1321, 1267, 1257, 1161, 1020, 947, 785 cm^−1^; ^1^H NMR (CDCl_3_, 500 MHz) *δ* 1.48-1.61 (1H, m, 4-H_pip_), 1.64–1.80 (3H, m, 4-H_pip_ + 5-H_2pip_), 2.02-2.13 (1H, m, 3-H_pip_), 2.39 (3H, s, CH_3_), 2.39–2.46 (1H, m, 3-H_pip_), 3.00–3.11 (1H, m, 6-H_pip_), 4.11–4.20 (1H, m, 6-H_pip_), 5.74–5.82 (1H, m, 2-H_pip_), 7.22 (1H, s, 4-H_isox_), 7.24 (2H, d, *J* = 8.0 Hz, 3,5-H_Ar_), 7.30 (1H, dd, *J* = 8.0, 4.7 Hz, 5-H_Py_), 7.56 (2H, d, *J* = 8.8 Hz, 2,6-H_Ar_), 7.64 (1H, d, *J* = 8.0 Hz, 4-H_Py_), 8.51 (1H, d, *J* = 4.4 Hz, 6-H_Py_), 8.61 (1H, d, *J* = 1.8 Hz, 2-H_Py_), 9.37 (s, C(O)NH); ^13^C NMR (CDCl_3_, 125 MHz) *δ* 19.40 (4-CH_2pip_), 21.63 (CH_3_), 25.65 (5-CH_2pip_), 27.88 (3-CH_2pip_), 41.45 (6-CH_2pip_), 51.86 (2-CH_pip_), 94.39 (4-CH_isox_), 123.93 (5-CH_Py_), 125.70 (2,6-CH_Ar_), 129.84 (3,5-CH_Ar_), 135.16 (4-CH_Py_), 148.04 (6-CH_Py_), 148.32 (2-CH_Py_); 124.86, 135.58, 140.83, 154.52, 160.91, 169.63 (6C_quater_); MS *m*/*z* (*I*_rel_, %) 363.20 [M+H]^+^ (100); Anal. calcd. for C_21_H_22_N_4_O_2_ (362.43): C, 69.83; H, 6.28; N, 15.31%; Found: C, 69.59; H, 6.12; N, 15.46%.

### 3.2. In Vitro Biological Assays

Cell lines. The work was carried out on C6 glioma cell lines (rat) from the collection of the Republican Research and Practical Center for Epidemiology and Microbiology (RRPCEM, Minsk, Belarus).

Sample preparation. Test samples were dissolved in dimethyl sulfoxide (DMSO) to a concentration of 0.1 M. Then the resulting solution was diluted in an isotonic solution (0.9% sodium chloride solution) to a concentration of compounds of 2000 μM.

Solutions of the test compounds were added to the wells with cells in a volume of 10% of the total volume of the culture medium (in a ratio of 1 (test compounds): 9 (medium with cells)). The final concentration of the test compounds was 200 μM, DMSO was 0.2%.

Antitumor drugs: cyclophosphamide (RUPE “Belmedpreparaty”, Minsk, Belarus), fluorouracil (RUPE “Belmedpreparaty”, Minsk, Belarus), etoposide (Fresenius Kabi, Bad Homburg, Germany), ribomustine (active substance—Bendamustine, Haupt Pharma Wolfratshausen GmbH, Bad Homburg, Germany), cisplatin (Pharmachemie B.V., Haarlem, The Netherlands).

Conducting experiments. Cells were seeded in wells of 96-well plates (Costar, Corning Inc., Corning, NY, USA) in DMEM (Merck KGaA, Darmstadt, Germany), supplemented with 10% fetal bovine serum (Sigma, St. Louis, MO, USA) and antibiotics (penicillin, streptomycin and neomycin, Biological Industries Beit Haemek, Israel). The test compounds were added to the wells at a final concentration of 200 μM, and/or antitumor drugs at a final concentration of 5 and 50 μM. In the control, instead of the tested compounds, a solvent dimethyl sulfoxide (DMSO), diluted in 0.9% sodium chloride, at a final concentration of 0.2%, was added. The number of samples in each series was n = 16 when studying the effects of **1**, **2**, **3**, and n = 8 when studying the effects of combined use of **1**, **2**, **3** with antitumor drugs. Cells were cultured for 48 h at 37 °C and 5% CO_2_. Then the cells were removed from the plate and cell samples were prepared. To assess cell viability, cells were stained with 7-aminoactinomycin (7-AAD) or propidium iodide. To determine the total number of cells and the number of viable cells in the samples (as an end point characterizing the antitumor effect), FLOW-COUNT™ reference fluorospheres (Beckman Coulter, Inc., Brea, CA, USA) were used according to the manufacturer’s instructions. Cellular samples were analyzed using a BD FACSCanto II flow cytometer (Becton Dickinson, Franklin Lakes, NJ, USA). To evaluate the effect of the combination against a tenfold increase in the dosage of the drug, a concentration of 5 μM was taken where a weak effect and low toxicity would appear, and this was increased to 50 μM for evaluating the effect of the drug–adjuvant combination against a tenfold increase in the drug dosage.

Statistical processing. The resulting digital material was processed by methods of variation statistics using the Excel 2016 and Statistica 7 software packages. The data are presented as the mean and its standard error. Differences between series were considered significant at a significance level of *p* < 0.05, according to Student’s *t* test.

### 3.3. Quantum Chemical Methods

The Gaussian 16 package software was used for DFT calculation, and for the visualization of the results of the DFT calculation, Gauss View 6.0.16 software was used. The calculations were performed within the framework of the DFT-D3 dispersion correction method [17], which, as shown in a number of works [18,19,20], is successfully used to take into account the inter- and intramolecular long-range dispersion interactions. Becke’s three-parameter exchange functional [21], in combination with the Lee et al. correlation functional [22] (B3LYP), were used. This was because this functional gives a high accuracy compared to experimental values for structures with intermolecular interactions, as demonstrated in [23,24].

Dunning’s correlation consistent polarized valence double zeta basis set with adding diffuse functions aug-cc-pVDZ was used. Diffuse functions were added for describing long-range interactions, such as Van der Waals forces, and noncovalent interactions, such as hydrogen bonding [25,26,27]. The LANL2DZ (Los Alamos National Laboratory 2 double zeta) basis set with effective core potential (ECP) was used for Pt [28].

The polarizable continuum model (PCM solvent is considered as a continuous dielectric medium) was used for solvent phase calculations [29]. The PCM model implements a self-consistent reaction field (SCRF) approach and defines solvent polarization in terms of electrostatic potential. The following discussions are based on this method, if not noted otherwise. No symmetric constraints were imposed during geometrical optimizations. The energy minima were identified by subsequent frequency calculations.

### 3.4. Molecular Docking

Molecular docking software packages AutoDock/Vina 4.2.6 and CHIMERA 1.16 were employed to calculate the protein–ligand binding interactions. The 3D crystal structure of the mouse thymidylate synthase (TS) was downloaded from the RCSB Protein Data Bank (PDB ID: 5FCT, 1.55 Å resolution). The protein was parameterized using AMBER f14SB force field, and the Gasteiger charges and hydrogen atoms were added to the examined receptor with native ligands. All water molecules and cofactors were removed before docking. The binding pockets of protein were identified using the CASTp package (http://cast.engr.uic.edu, accessed on 15 March 2024), and the grid box had the following parameters: ΔX 24 Å, ΔY 32 Å, ΔZ 29 Å centered at (5.0; −5.5; −5.0). The docking calculations were performed with exhaustiveness of 8 and an energy range equal to 3 kcal/mol.

**Figure 7:**
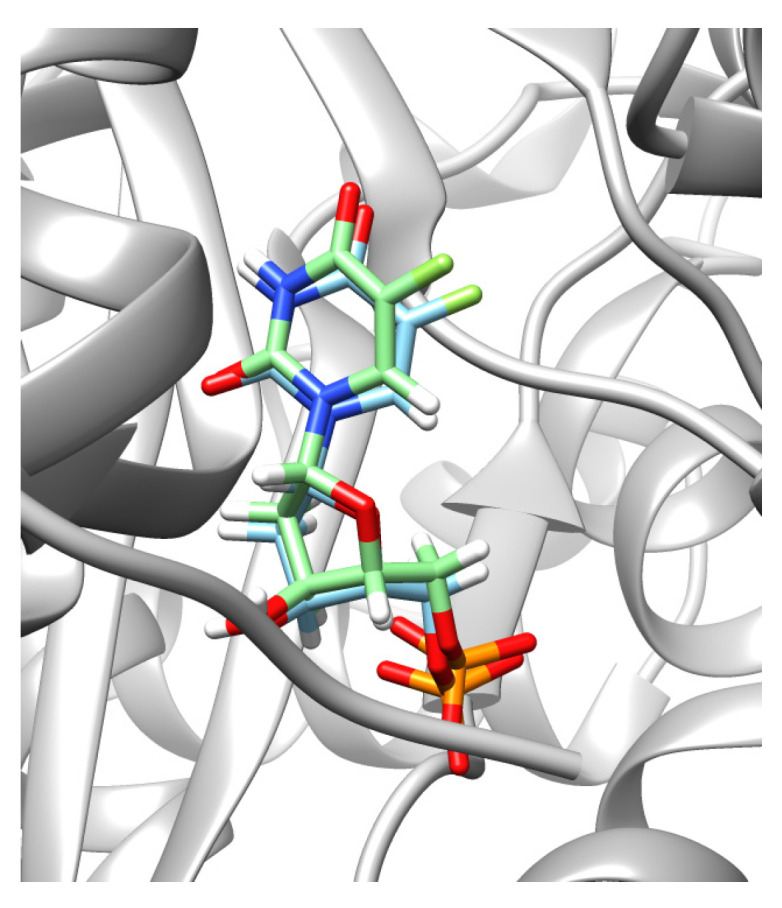
Location of native co-crystallized (blue) and docked (green) ligands.

The validation of the procedure involved docking the X-ray ligand conformation rigidly to the active site following the protein preparation step. The 3D structure of UFP ligand was isolated from the crystal UFP-C2F-TS complex. The results of a comparative analysis of complexes of UFP inhibitor with TS, constructed using the methods of X-ray crystallography and molecular docking (Figure 7), indicate that the calculation protocol we used provided sufficient accuracy (RMSD < 2 Å, Figure 7) in predicting the orientation of the ligand in the catalytic site of the enzyme. This suggests that the data we obtained for the isoxazolylurea adequately describe the main geometric and energetic characteristics of their complexes with thymidylate synthase. Intermolecular interactions between ligand and receptor protein were analyzed by BIOVIA Discovery Studio Visualizer 2024.

## 4. Conclusions

Taking into account the combination of theoretical calculations, molecular docking and test results, we can conclude:

In vitro tests of alkaloid-based isoxazolylureas on the C6 rat glioma model showed that a synergistic effect was observed to varying degrees for all studied compounds when using binary mixtures with alkylating chemotherapy drugs cisplatin and ribomustine, whereas antagonism and the tendency to antagonism are noted for the remaining drugs, except for the combination with fluorouracil, where conflicting results were observed.

The clear antagonistic effect of the combination of urea with cyclophosphamide, fluorouracil and etoposide, resulting in a 2–3 fold decrease of antiproliferative action, can be explained based on the results of quantum chemical modeling of the spatial structure of the adjuvants. The structure of isoxazolylurea 2 differed from the other two in its most linear spatial arrangement, which could result in the varying degrees of conjugate stability. In addition, analysis of FMO localization in fluorouracil conjugates showed that HOMO and LUMO, in contrast to cisplatin conjugates, were completely localized on isoxazole heterocycle or alkaloid fragments, but not on drug molecules. This means that the interaction with the electrophilic and nucleophilic regions of protein sites is realized mainly due to the adjuvant molecule.

Based on the molecular docking data, all isoxazolylureas demonstrated a good degree of binding to the thymidylate synthase protein site, forming an extensive system of residue interactions mainly due to the hydrophobic interactions of electron-rich π systems of isoxazole heterocycle and aromatic rings with amino acid residues. This probably confirms the possibility of competing binding processes with the thymidylate synthase site, but does not explain the synergy observed in the case of the composition of fluorouracil with anabasine isoxazolylurea 3. It cannot be ruled out that ureas could be multitarget ligands that attack other proteins as well, and observed discrepant behavior of fluorouracil compositions should instead be explained by varying degrees of stability of drug–adjuvant complexes with the formation of an inactive product.

Isothiazolylurea adjuvants shaped conjugates with cisplatin with non-covalent interactions between molecules due to hydrogen and van der Waals bonds generally forming more stable complexes than with fluorouracil. FMOs here were uniformly distributed between the adjuvant and the cisplatin molecule. This may indicate a coordinated mechanism of action cisplatin and the adjuvant in binary mixtures towards biological targets, which was confirmed by the experimentally observed synergistic effect.

The most pronounced synergistic effect was observed in the case of ribomustine mixtures with all synthesized compounds, which encourages further research of the dose-dependent characteristics for this composition.

## Figures and Tables

**Table 1:** Decrease in viable C6 rat glioma cell concentration (%) for antitumor drugs (c = 5 and 50 μM), compounds **1-3** (c = 200 μM) and their composition.

Decrease in Viable C6 Rat Glioma Cell Concentration, %		Compound, c = 200 µM (0.2% DMSO)							
		1		2		3		Drug, c = 50 µM	
Drug. c = 5 µM		18.7 ± 5.8		24.7 ± 5.8		8.2 ± 5.9			
**CP**	26.6 ± 4.1	41.0 ± 3.2	Σ 45.3	22.2 ± 1.5	Σ 51.3	19.3 ± 3.1	Σ 34.8	**CP**	33.2 ± 3.7
**FU**	7.2 ± 8.2	24.0 ± 6.1	Σ 25.9	10.2 ± 9.6	Σ 31.9	23.2 ± 3.3	Σ 15.4	**FU**	42.3 ± 4.1
**ET**	28.20 ± 4.1	45.0 ± 2.3	Σ 46.9	20.9 ± 4.8	Σ 52.9	34.8 ± 2.2	Σ 36.4	**ET**	44.2 ± 3.5
**CPt**	25.3 ± 5.9	47.4 ± 5.8	Σ 44.0	65.0 ± 2.8	Σ 50.0	45.5 ± 10.1	Σ 33.5	**CPt**	93.0 ± 0.3
**RM**	3.2 ± 10.3	33.9 ± 1.9	Σ 21.9	57.6 ± 2.1	Σ 27.9	21.3 ± 9.6	Σ 11.4	**RM**	35.7 ± 1.3

Σ is the sum of the effects of a single compound and drug.

**Table 2:** Energy characteristics of the conjugates formation process from the initial components **1-3**, **FU**, **CPt**.

Conjugate	∆E_f_, a.e.	∆E_f_, kcal/mol
1+FU	−0.009871226	−6.19
2+FU	−0.026896256	−16.88
3+FU	−0.018223976	−11.44
1+CPt	−0.03977506	−24.96
2+CPt	−0.02215013	−13.90
3+CPt	−0.03375495	−21.18

**Table 3:** Theoretical electronic parameters (energies of HOMO and LUMO, energy gap ∆E,) and global reactivity descriptors for compounds **1-3**, flourouracil, cisplatin and their conjugates.

	E_HOMO_, eV	E_LUMO_, eV	∆E, eV	μ (eV)	χ (eV)	η (eV)	S (eV^−1^)	ω (eV)	D, Db
**1**	−5.7689	−1.5192	4.2496	−3.6441	3.6441	2.1248	0.2353	3.1248	10.24
**2**	−5.9797	−1.4343	4.5454	−3.7070	3.7070	2.2727	0.2200	3.0232	5.24
**3**	−6.2992	−1.5160	4.7833	−3.9076	3.9076	2.3917	0.2091	3.1922	6.94
**FU**	−6.6562	−1.2798	5.3765	−3.9680	3.9680	2.6883	0.1860	2.9281	5.13
**1+FU**	−5.7653	−1.5241	4.2412	−3.6447	3.6447	2.1206	0.2358	3.1321	8.23
**2+FU**	−5.9923	−1.5347	4.4575	−3.7635	3.7635	2.2288	0.2243	3.1775	3.57
**3+FU**	−6.3055	−1.5206	4.7849	−3.9131	3.9131	2.3925	0.2090	3.2000	2.01
**CPt**	−6.5305	−1.7475	4.7830	−4.1390	4.1390	2.3915	0.2091	3.5817	15.94
**1+CPt**	−5.9642	−1.6504	4.3139	−3.8073	3.8073	2.1570	0.2318	3.3601	9.44
**2+CPt**	−5.9931	−1.6784	4.3147	−3.8358	3.8358	2.1574	0.2318	3.4100	19.75
**3+CPt**	−6.1966	−1.5720	4.6246	−3.8843	3.8843	2.3123	0.2162	3.2625	7.77

**Table 4:** Molecular docking analysis of binding **1-3**, **FU**, **FdUMP** with thymidylate synthase (PDB ID: 5FCT).

	Binding Energy, ΔG (kcal/mol)	Residues Interactions			
		Hydrogen Bond	Electrostatic Interactions	Hydrophobic Bond	Pi-Sulfur
**1**	−9.3	0	0	6 (LEU215, PHE74, PHE219, ILE102, TRP103)	0
**2**	−9.9	3 (ASP212, HIS250)	0	7 (PHE74, ILE102, LEU215, MET305, LEU186, CYS189)	1 (CYS189)
**3**	−10.0	2 (ASP212, GLY216)	3 (ARG44, GLU81, ASP212)	6 (ARG169, LEU186, MET305, TRP103, ILE102)	1 (CYS189)
**FU**	−5.1	8 (ASP212, GLN208, ASN220, GLY211, HIS190, CYS189)	40	0	1 (CYS189)
**FdUMP**	−9.5	15 (ASN220, ASP212, GLN208, GLN211, CYS189, SER210, ARG44, ARG169, ARG170, ARG209, TYR252)	8 (ARG44, ARG169, ARG209, ARG107)	0	1 (CYS189)

## Data Availability

The data presented in this study are available in the article or Supplementary Material.

## Supplementary Materials

The following supporting information can be downloaded at: https://www.mdpi.com/article/10.3390/molecules29143246/s1, NMR, MS, IR.
