# Supplementary material for: Alkaloid-Based Isoxazolylureas: Synthesis and Effect in Combination with Anticancer Drugs on C6 Rat Glioma Model Cells
Source: Molecules. 2024 Jul 9;29(14):3246. doi: 10.3390/molecules29143246 (PMC11278957; doi:10.3390/molecules29143246)
Supplement: Supplementary file 1 [file molecules-29-03246-s001.zip › IR-UV-MS.pdf]

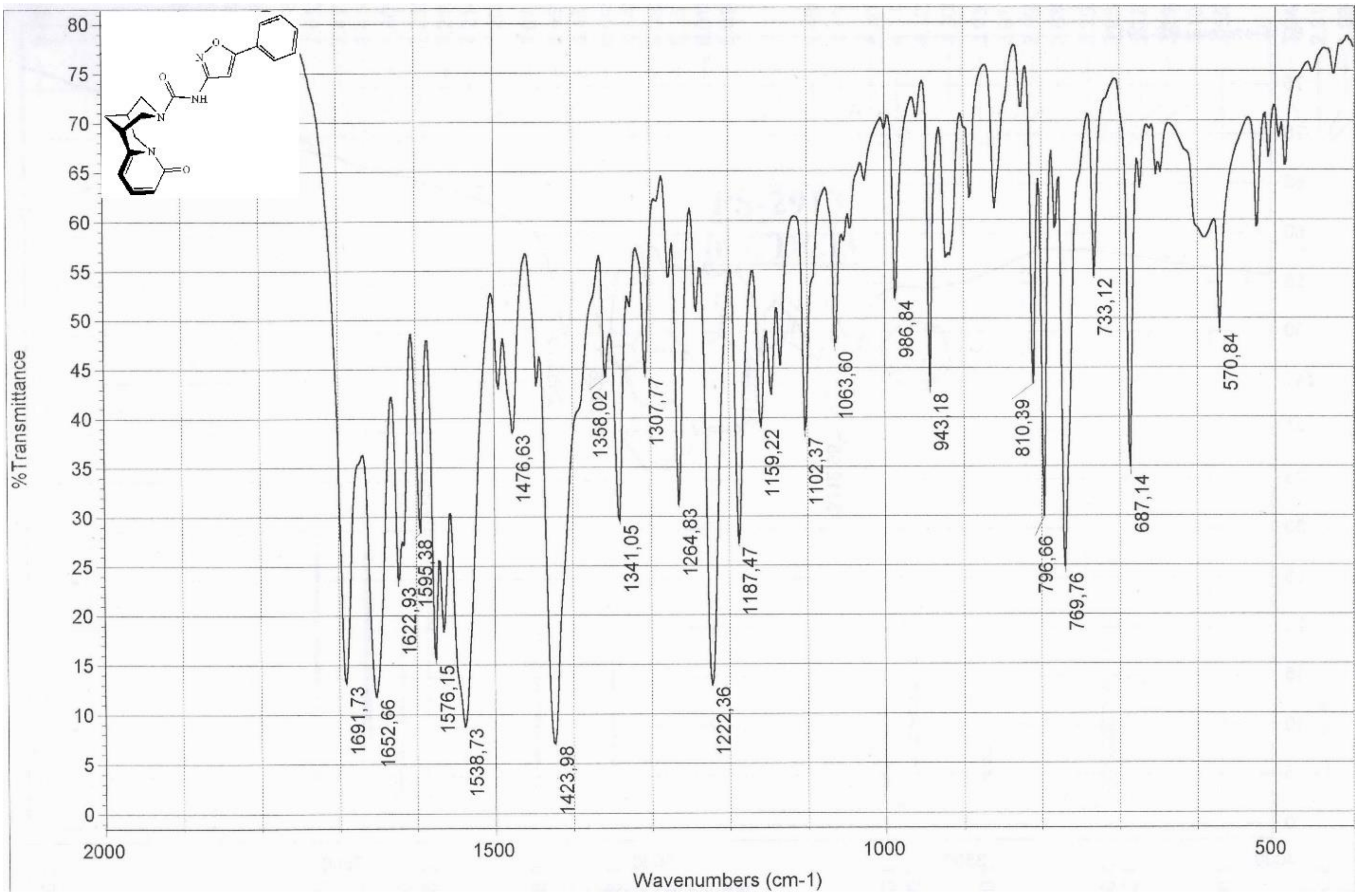
%Transmittance
Wavenumbers (cm-1)
1691,73
1652,66
1622,93
1595,38
1576,15
1538,73
1476,63
1423,98
1358,02
1341,05
1307,77
1264,83
1222,36
1187,47
1159,22
1102,37
1063,60
986,84
943,18
810,39
796,66
769,76
733,12
687,14
570,84
O
N
NH

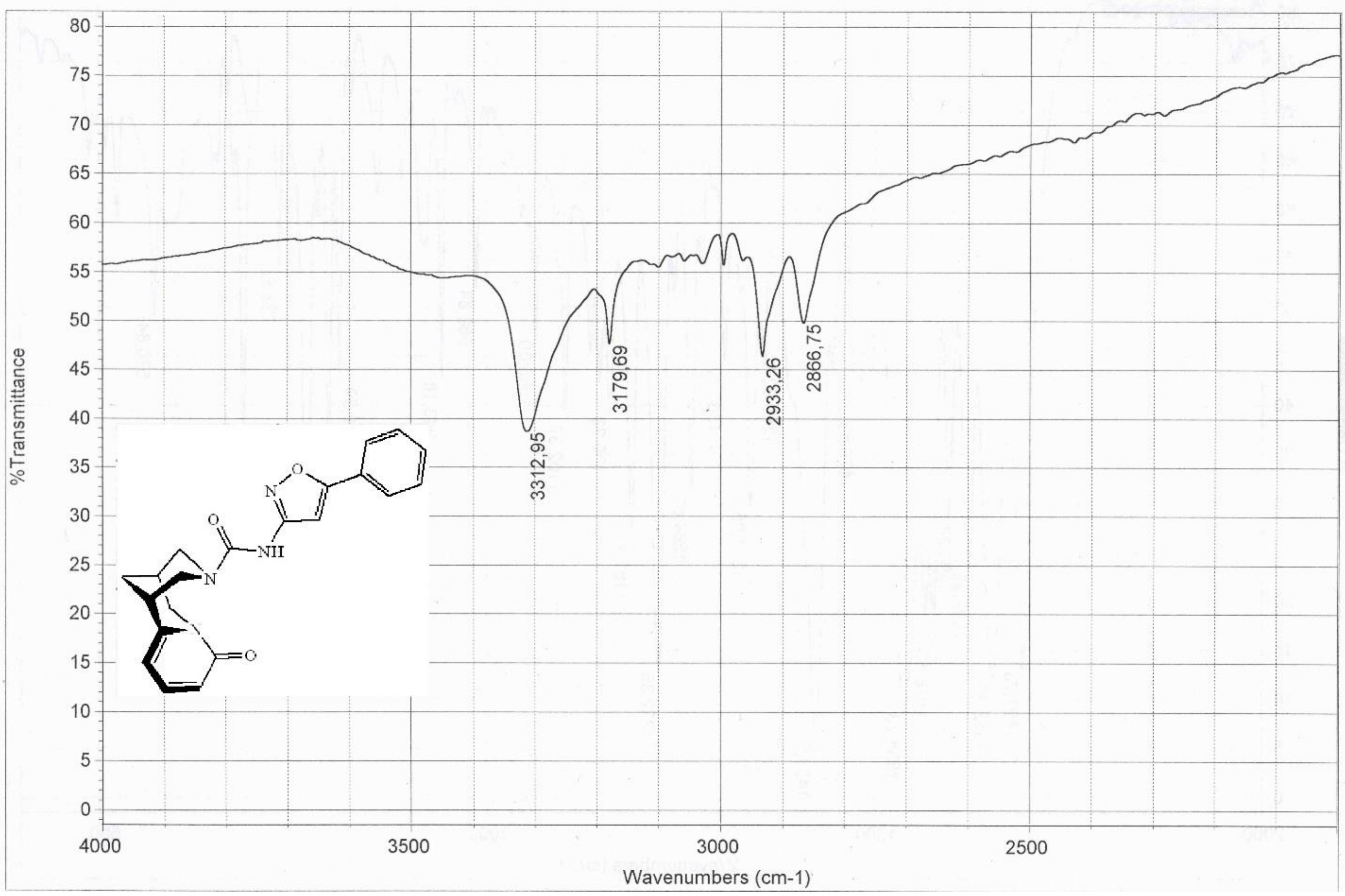
%Transmittance
Wavenumbers (cm-1)
3312,95
3179,69
2933,26
2866,75
O
N
NH

Qualitative Analysis Report

**Data Filename**	ps-2913_02.d	**Sample Name**	ps-2913
**Sample Type**	Sample	**Position**	Vial 2
**Instrument Name**	Instrument 1	**User Name**	
**Acq Method**	All_2021_kol 1-2.m	**Acquired Time**	4/19/2022 10:19:00 AM
**IRM Calibration Status**	Not Applicable	**DA Method**	Defaul1t.m
**Comment**			

**Sample Group**		**Info.**	
**Stream Name**	LC 1	**Acquisition SW Version**	6400 Series Triple Quadrupole 10.0 (127)

User Chromatograms

Fragmentor Voltage 135 **Collision Energy** 0 **Ionization Mode** ESI

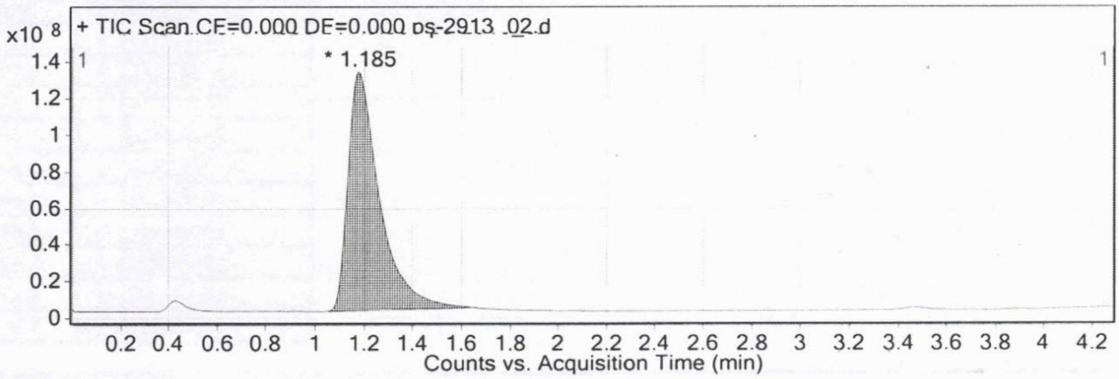

Integration Peak List

Peak	Start	RT	End	Height	Area	Area %
1	1,046	1,185	1,64	129278539,1	1170460751	100

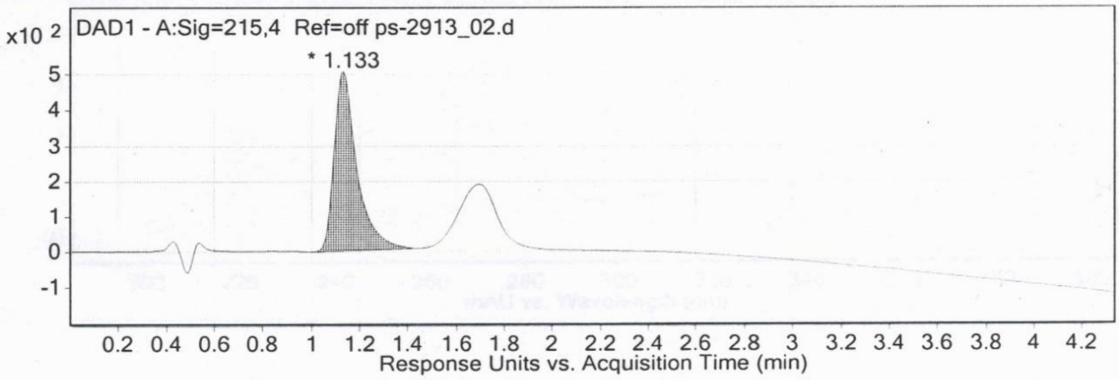

Integration Peak List

Peak	Start	RT	End	Height	Area	Area %
1	0,993	1,133	1,433	508,26	3318,61	100

User Spectra

Spectrum Source	Fragmentor Voltage	Collision Energy	Ionization Mode
Peak (1) in "+ TIC Scan"	135	0	ESI

Qualitative Analysis Report

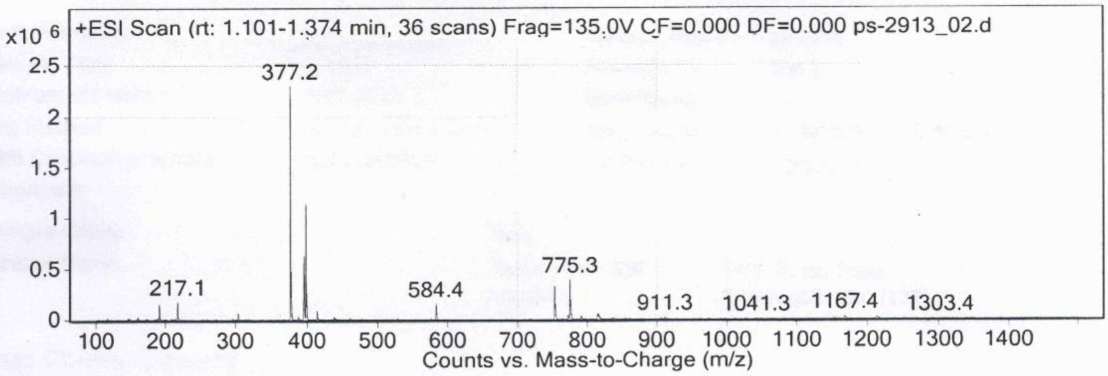

Peak List

m/z	z	Abund
217.1	1	177376.78
377.2	1	2301492.25
378.2	1	477573.78
396.3		631910.38
399.1	1	1147305.13
400.1	1	257379.94
753.3	1	331467.66
754.3	1	155543.52
775.3	1	392187.53
776.3	1	190853.75

Spectrum Source
Peak (1) in "DAD1 - A:Sig=215,4 Ref=off"

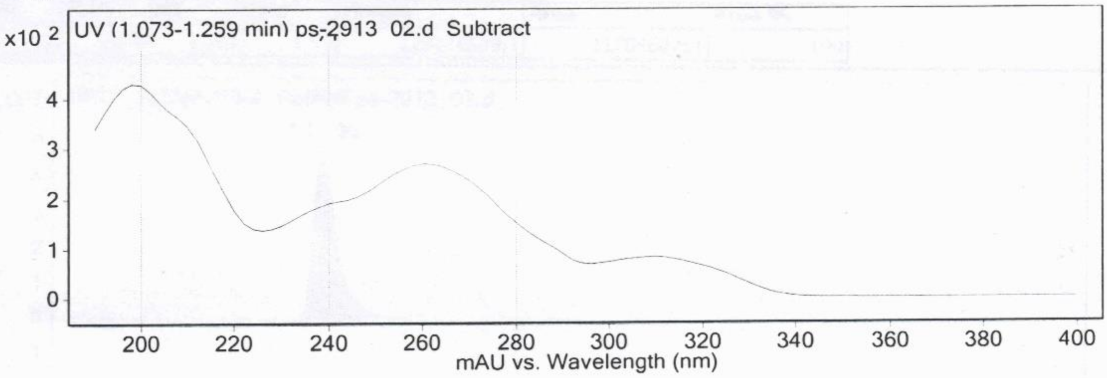

--- End Of Report ---

24.06.22 11:51:35 Page 1 of 1

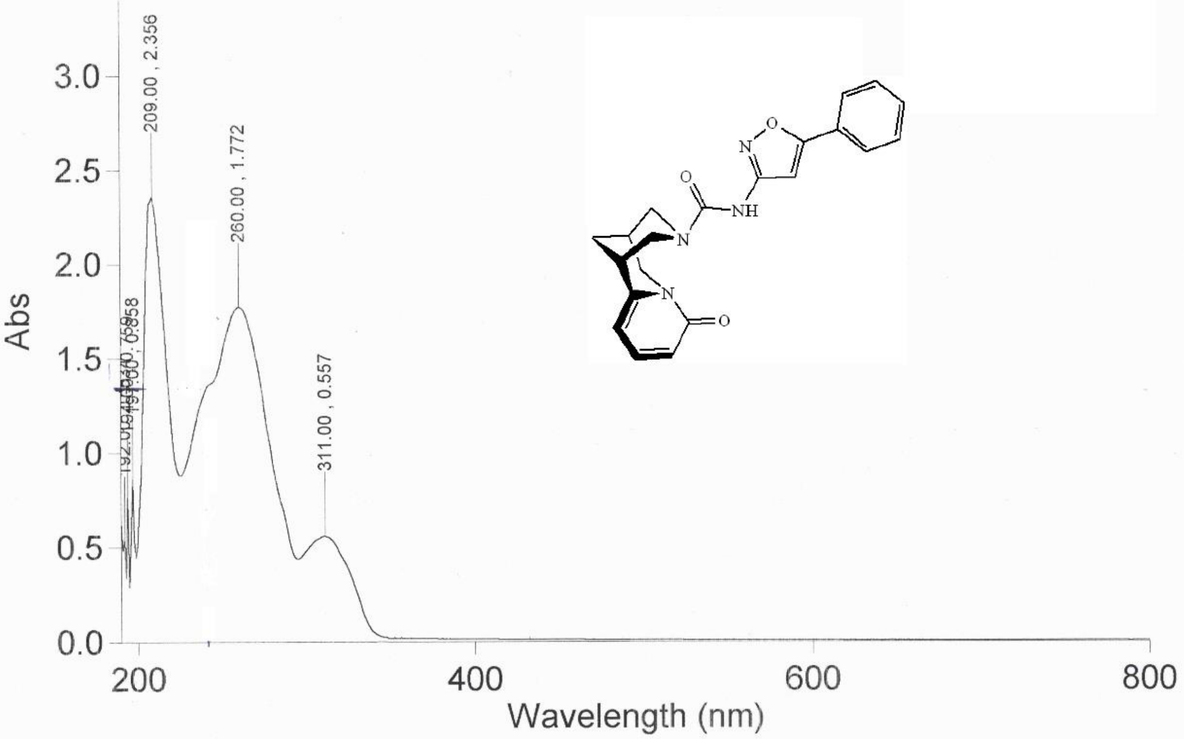

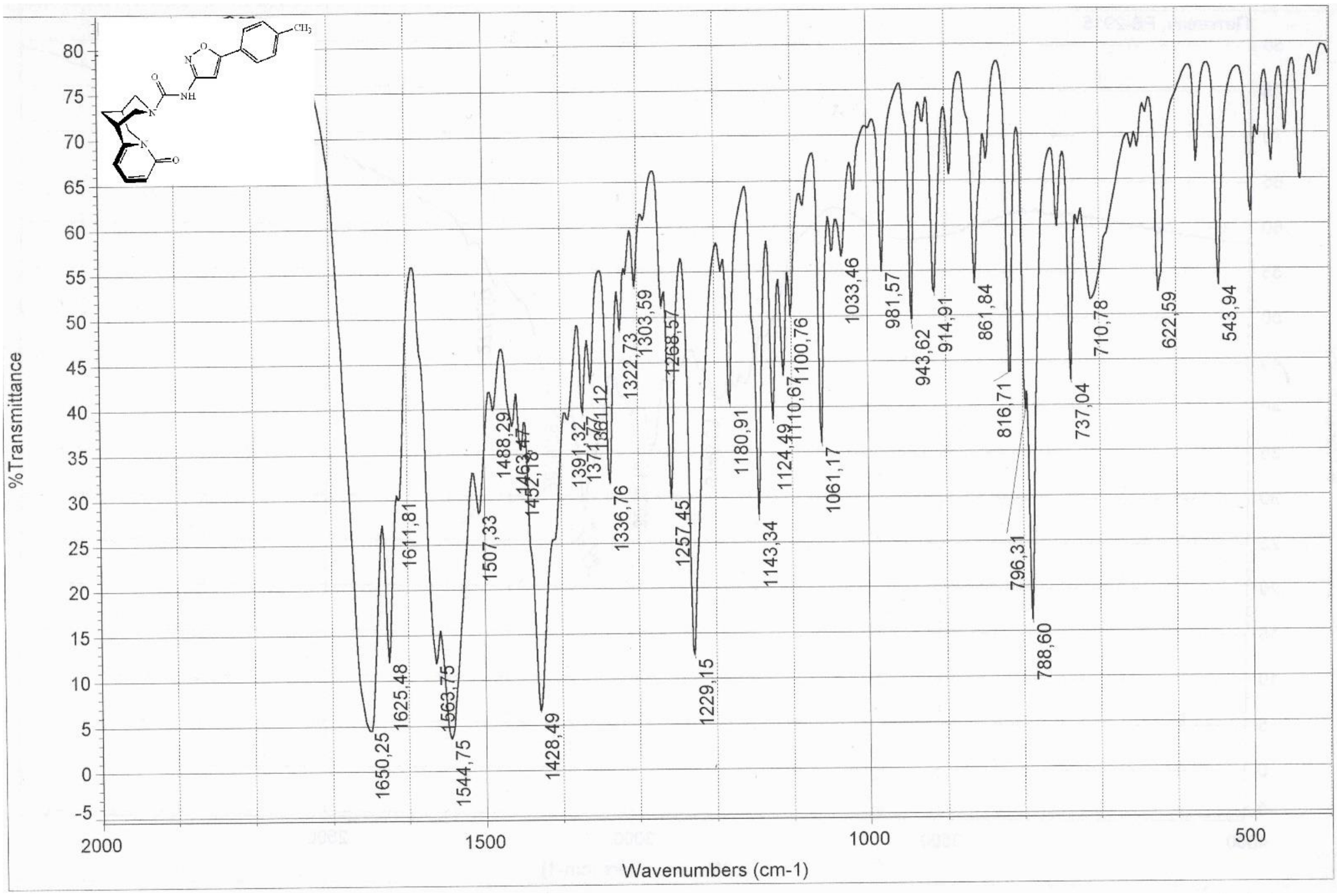
%Transmittance
Wavenumbers (cm-1)
1650,25
1625,48
1611,81
1563,75
1544,75
1507,33
1488,29
1463,47
1452,18
1428,49
1391,32
1361,12
1336,76
1322,73
1303,59
1268,57
1257,45
1229,15
1180,91
1143,34
1124,49
1110,67
1100,76
1061,17
1033,46
981,57
943,62
914,91
861,84
816,71
796,31
788,60
737,04
710,78
622,59
543,94

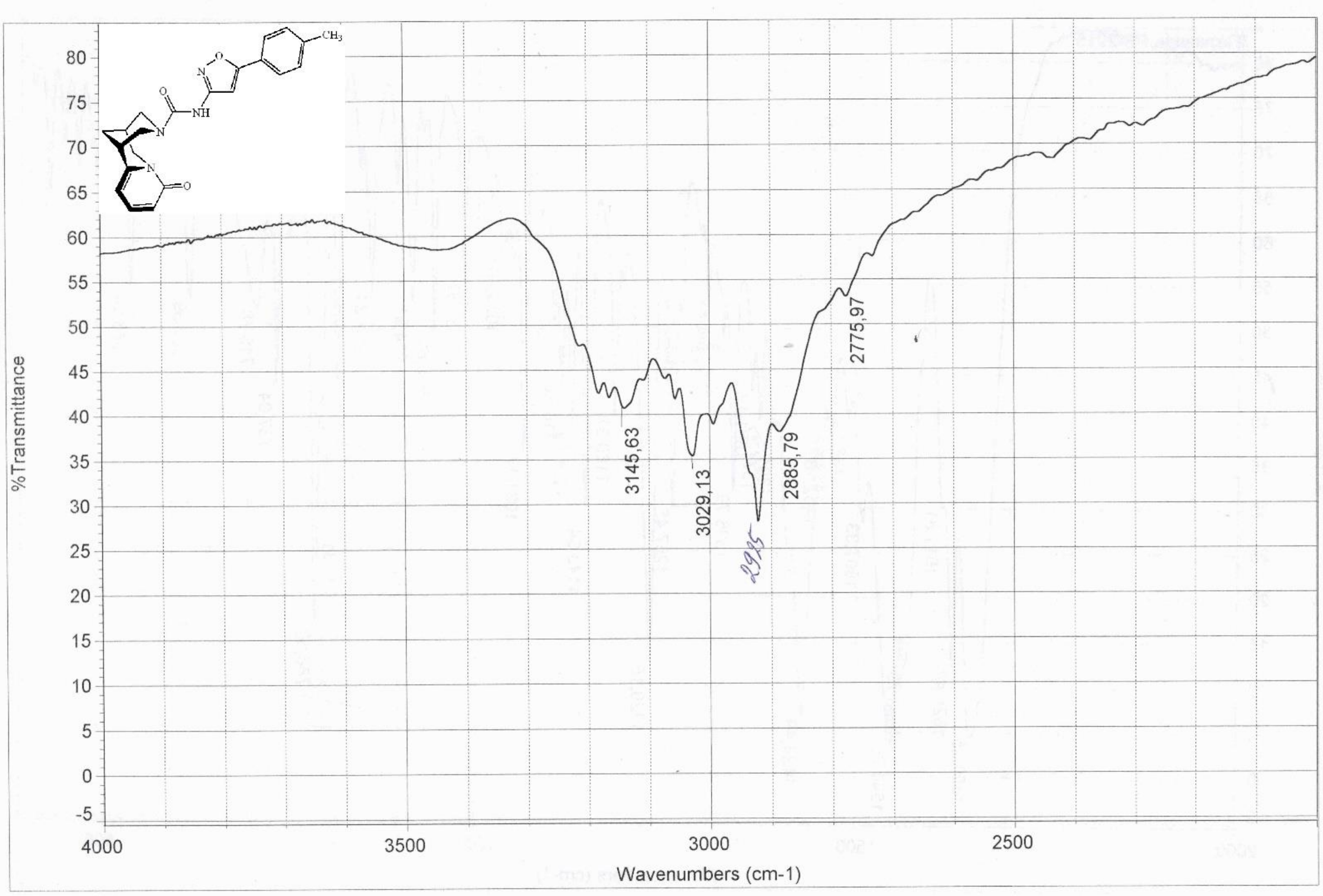
%Transmittance
Wavenumbers (cm-1)
3145,63
3029,13
2925
2885,79
2775,97

Qualitative Analysis Report

**Data Filename**	ps-2915_01.d	**Sample Name**	ps-2915
**Sample Type**	Sample	**Position**	Vial 2
**Instrument Name**	Instrument 1	**User Name**	
**Acq Method**	All_2021_kol 1-2.m	**Acquired Time**	4/19/2022 11:00:01 AM
**IRM Calibration Status**	Not Applicable	**DA Method**	Defaul1t.m
**Comment**			

**Sample Group**		**Info.**	
**Stream Name**	LC 1	**Acquisition SW Version**	6400 Series Triple Quadrupole 10.0 (127)

User Chromatograms

Fragmentor Voltage 135 **Collision Energy** 0 **Ionization Mode** ESI

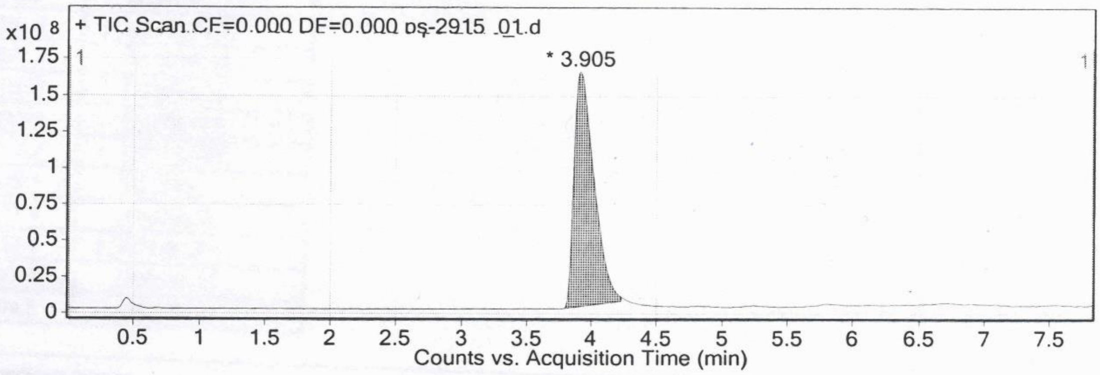

Integration Peak List

Peak	Start	RT	End	Height	Area	Area %
1	3,78	3,905	4,225	161647536,1	1731559915	100

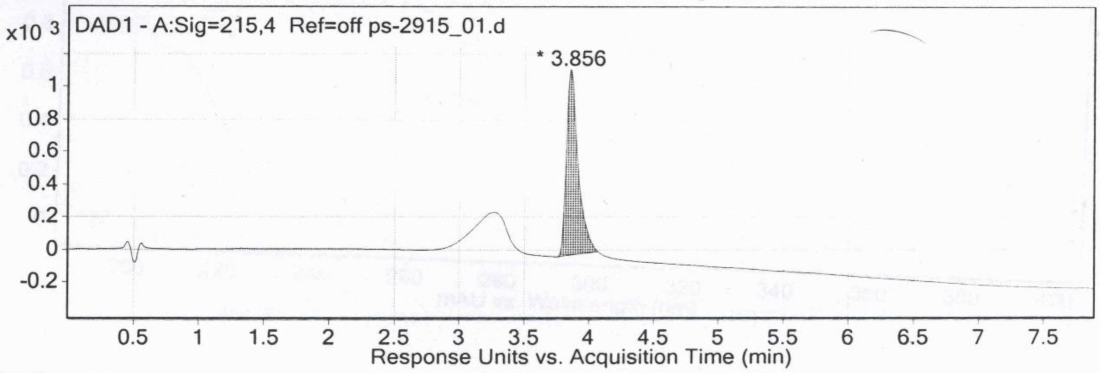

Integration Peak List

Peak	Start	RT	End	Height	Area	Area %
1	3,763	3,856	4,069	1140,51	7176,38	100

User Spectra

Spectrum Source	Fragmentor Voltage	Collision Energy	Ionization Mode
Peak (1) in "+ TIC Scan"	135	0	ESI

Qualitative Analysis Report

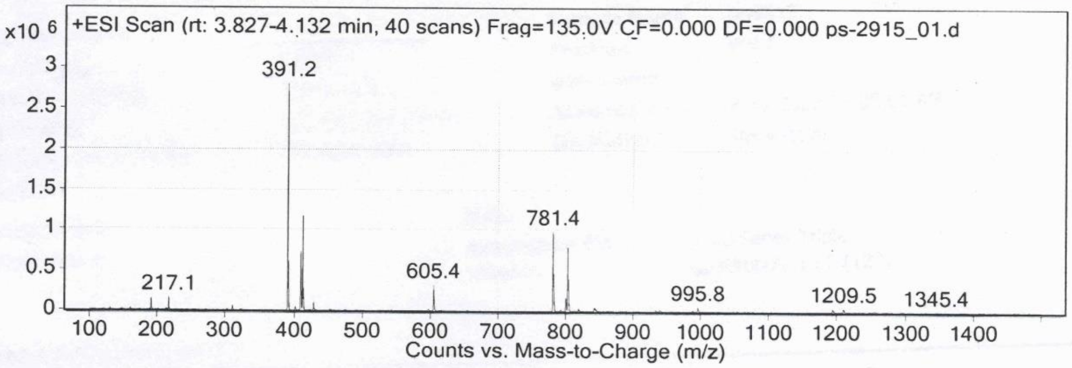

Peak List

m/z	z	Abund
391.2	1	2806674.25
392.2	1	612238.44
410.3		735016.75
413.1		1170176.5
414.2	1	277797.84
605.4		322778.5
781.4	1	989784.69
782.4	1	470381.84
803.4		790936.81
804.3		391555.41

Spectrum Source

Peak (1) in "DAD1 - A:Sig=215,4 Ref=off"

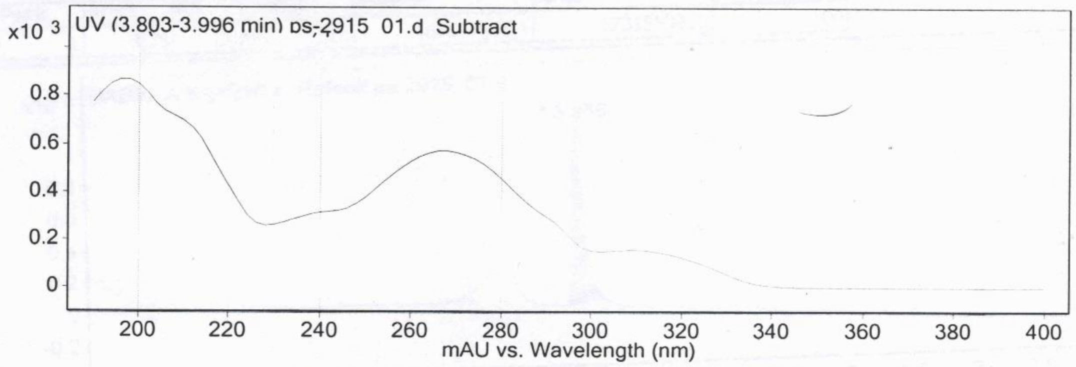

--- End Of Report ---

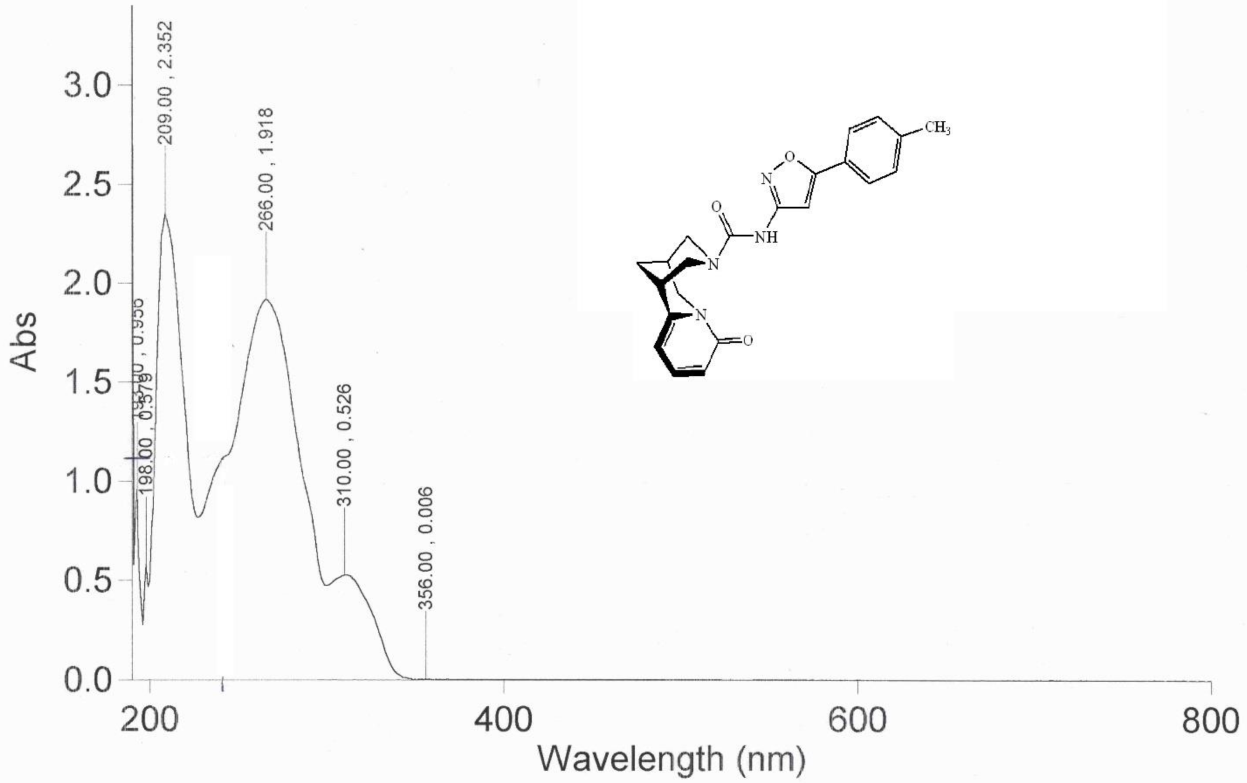
3.0
2.5
2.0
1.5
1.0
0.5
0.0
Abs
209.00 , 2.352
266.00 , 1.918
310.00 , 0.526
356.00 , 0.006
200
400
600
800
Wavelength (nm)
CH3
O
N
O
NH
N
N
O

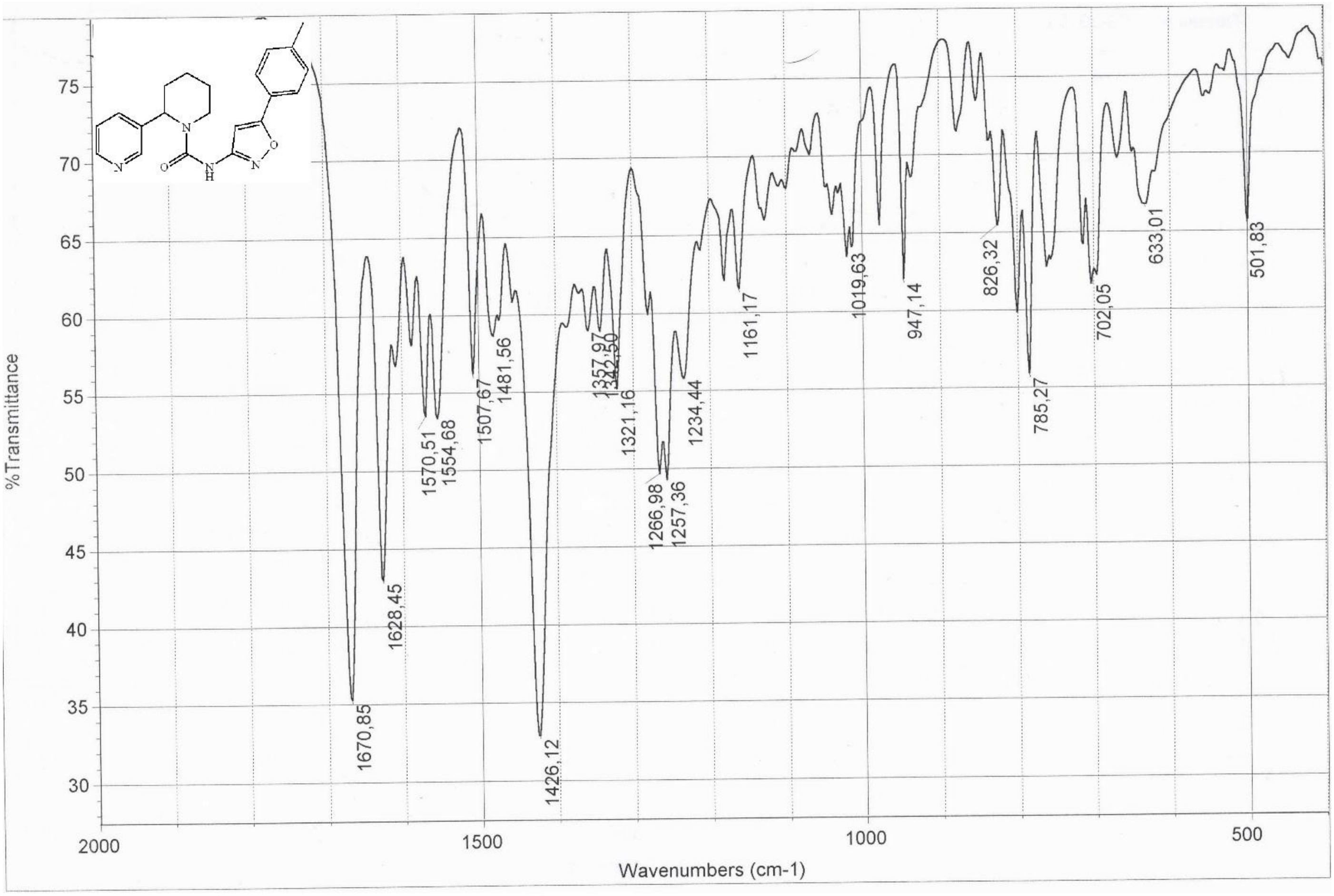

%Transmittance
Wavenumbers (cm-1)
1670,85
1628,45
1570,51
1554,68
1507,67
1481,56
1426,12
1357,97
1321,16
1266,98
1257,36
1234,44
1161,17
1019,63
947,14
826,32
785,27
702,05
633,01
501,83

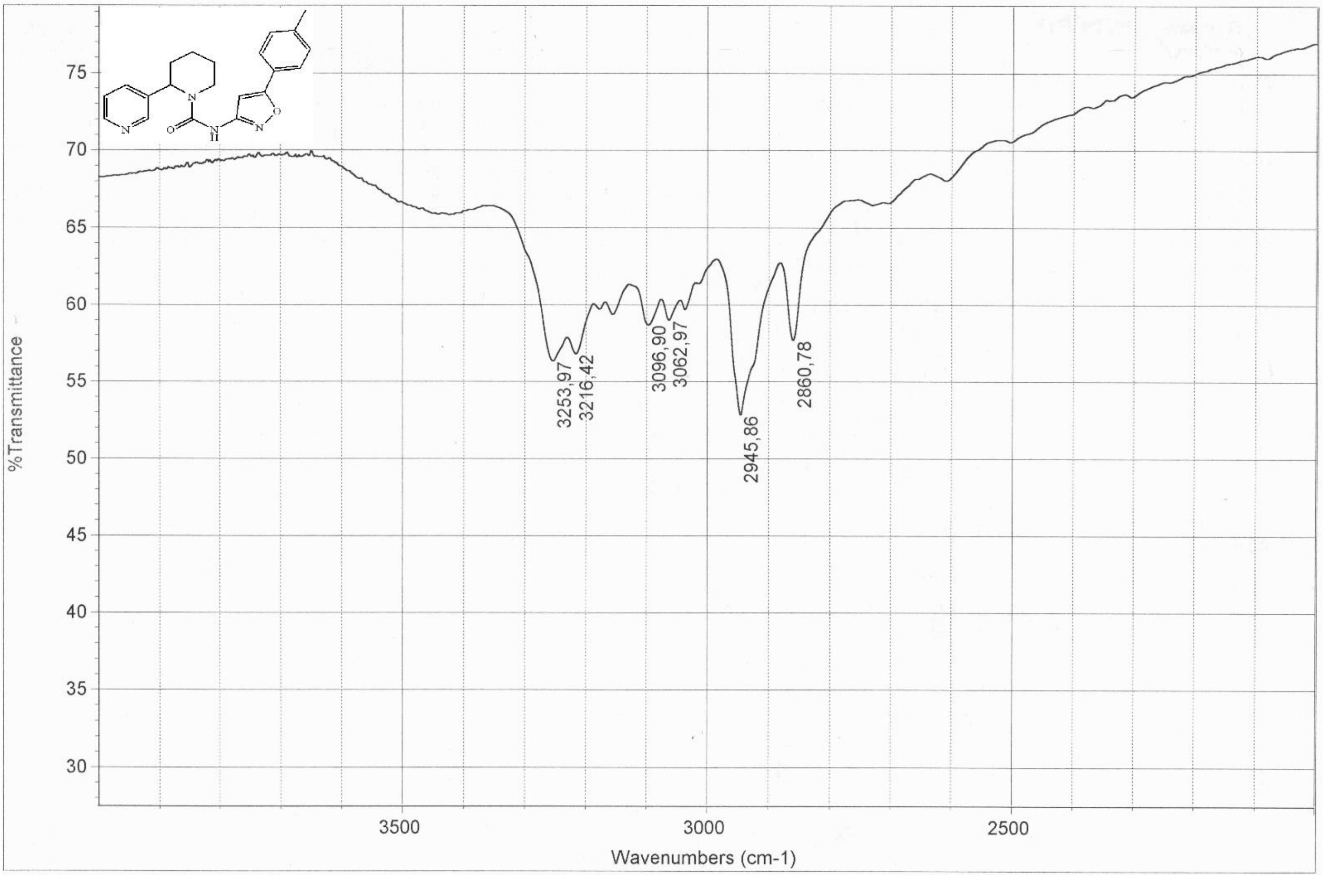

%Transmittance
Wavenumbers (cm-1)
3253,97
3216,42
3096,90
3062,97
2945,86
2860,78

Qualitative Analysis Report

**Data Filename**	ps2916_01.d	**Sample Name**	ps2916
**Sample Type**	Sample	**Position**	Vial 2
**Instrument Name**	Instrument 1	**User Name**	
**Acq Method**	All_2021_kol 1-2.m	**Acquired Time**	6/13/2022 10:45:38 AM
**IRM Calibration Status**	Not Applicable	**DA Method**	Defaul1t.m
**Comment**			

**Sample Group**		**Info.**	
**Stream Name**	LC 1	**Acquisition SW Version**	6400 Series Triple Quadrupole 10.0 (127)

User Chromatograms

Fragmentor Voltage 135 **Collision Energy** 0 **Ionization Mode** ESI

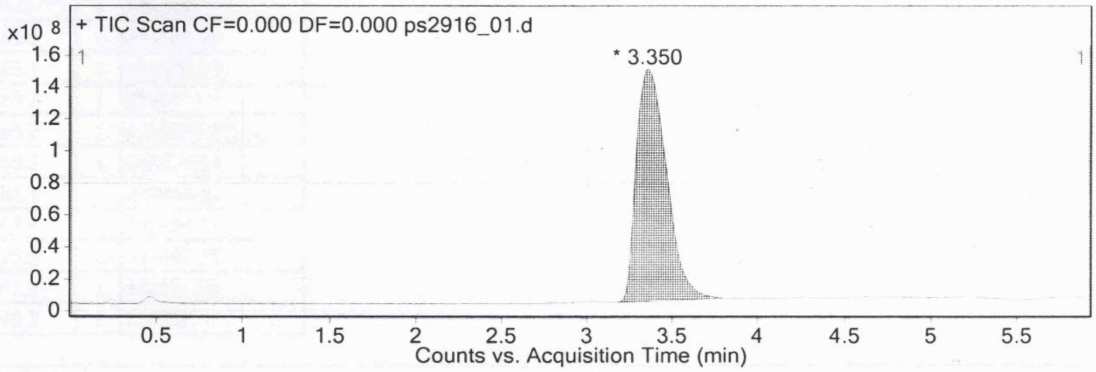

Integration Peak List

Peak	Start	RT	End	Height	Area	Area %
1	3,171	3,35	3,796	144786918,8	1793838029	100

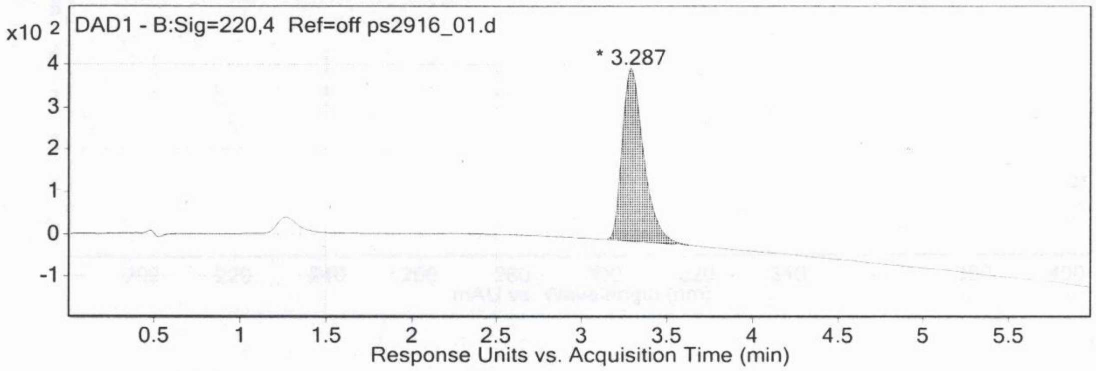

Integration Peak List

Peak	Start	RT	End	Height	Area	Area %
1	3,14	3,287	3,633	409,19	3617,65	100

User Spectra

Spectrum Source	Fragmentor Voltage	Collision Energy	Ionization Mode
Peak (1) in "+ TIC Scan"	135	0	ESI

Qualitative Analysis Report

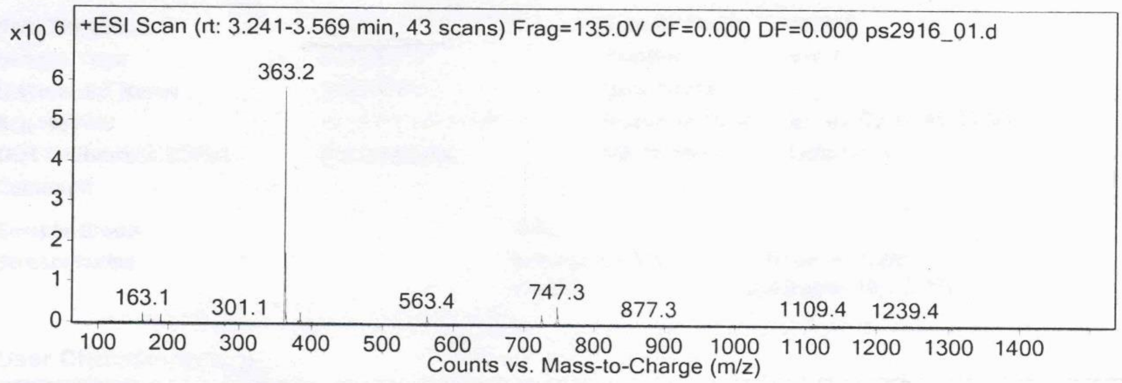

Peak List

m/z	z	Abund
163.1	1	239660.28
189.1	1	194080.86
363.2	1	5859971.5
364.2	1	1733569.63
365.2	1	205814.53
385.1	1	248945.06
563.4		199494.77
725.3	1	244442.75
747.3	1	442151.88
748.3	1	216982.42

Spectrum Source

Peak (1) in "DAD1 - B:Sig=220,4 Ref=off"

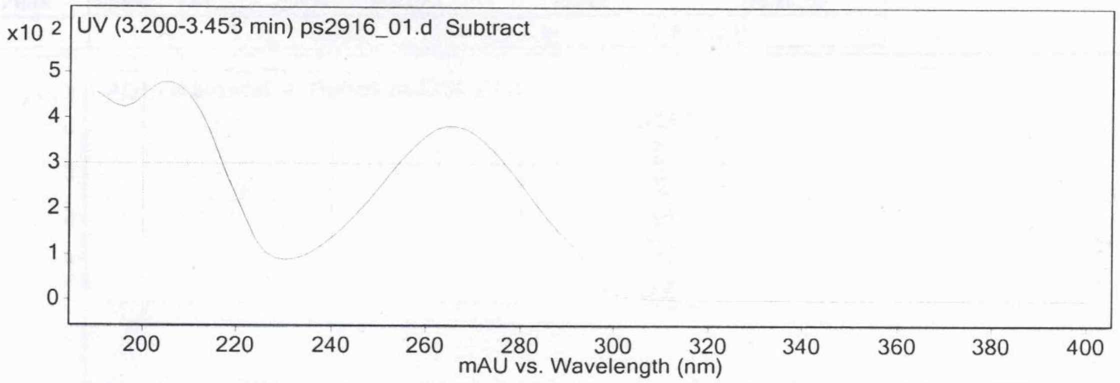

--- End Of Report ---

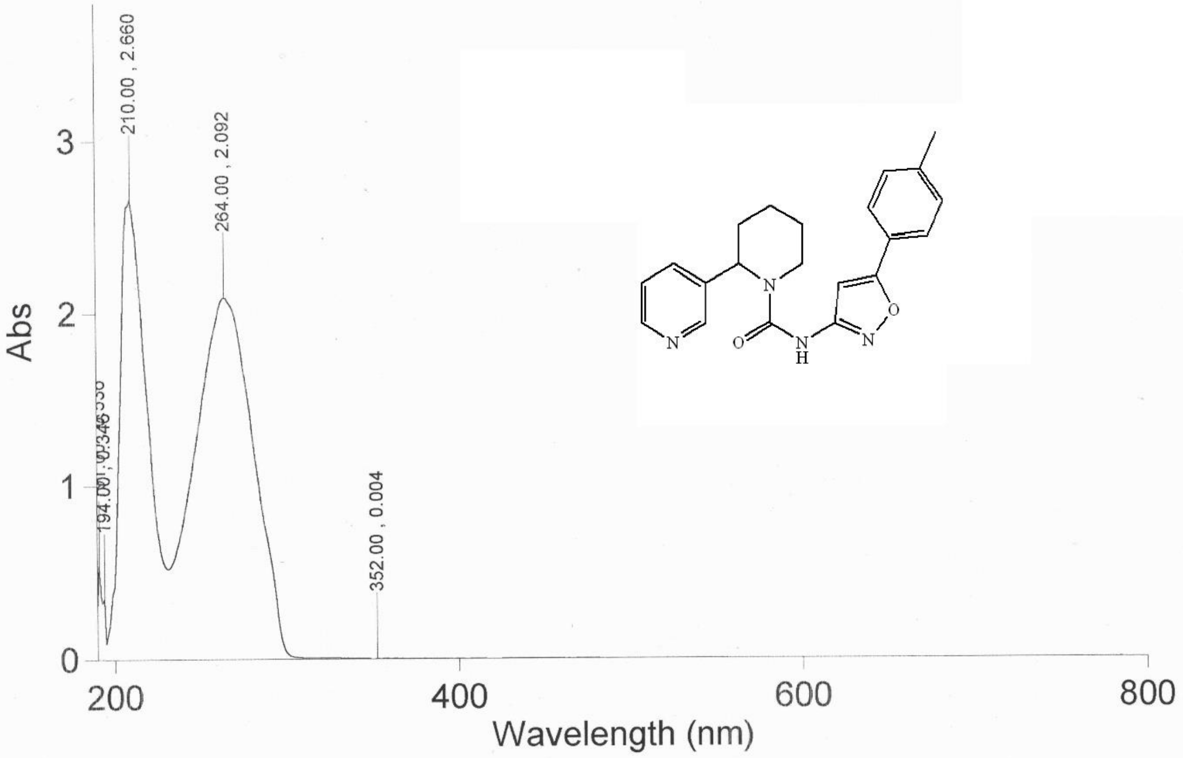
210.00 , 2.660
264.00 , 2.092
352.00 , 0.004
Abs
0
1
2
3
200
400
600
800
Wavelength (nm)
N
N
O
N
H
N
O
