# Supplementary figures and images for: Alkaloid-Based Isoxazolylureas: Synthesis and Effect in Combination with Anticancer Drugs on C6 Rat Glioma Model Cells
Source: Molecules. 2024 Jul 9;29(14):3246. doi: 10.3390/molecules29143246 (PMC11278957; doi:10.3390/molecules29143246)

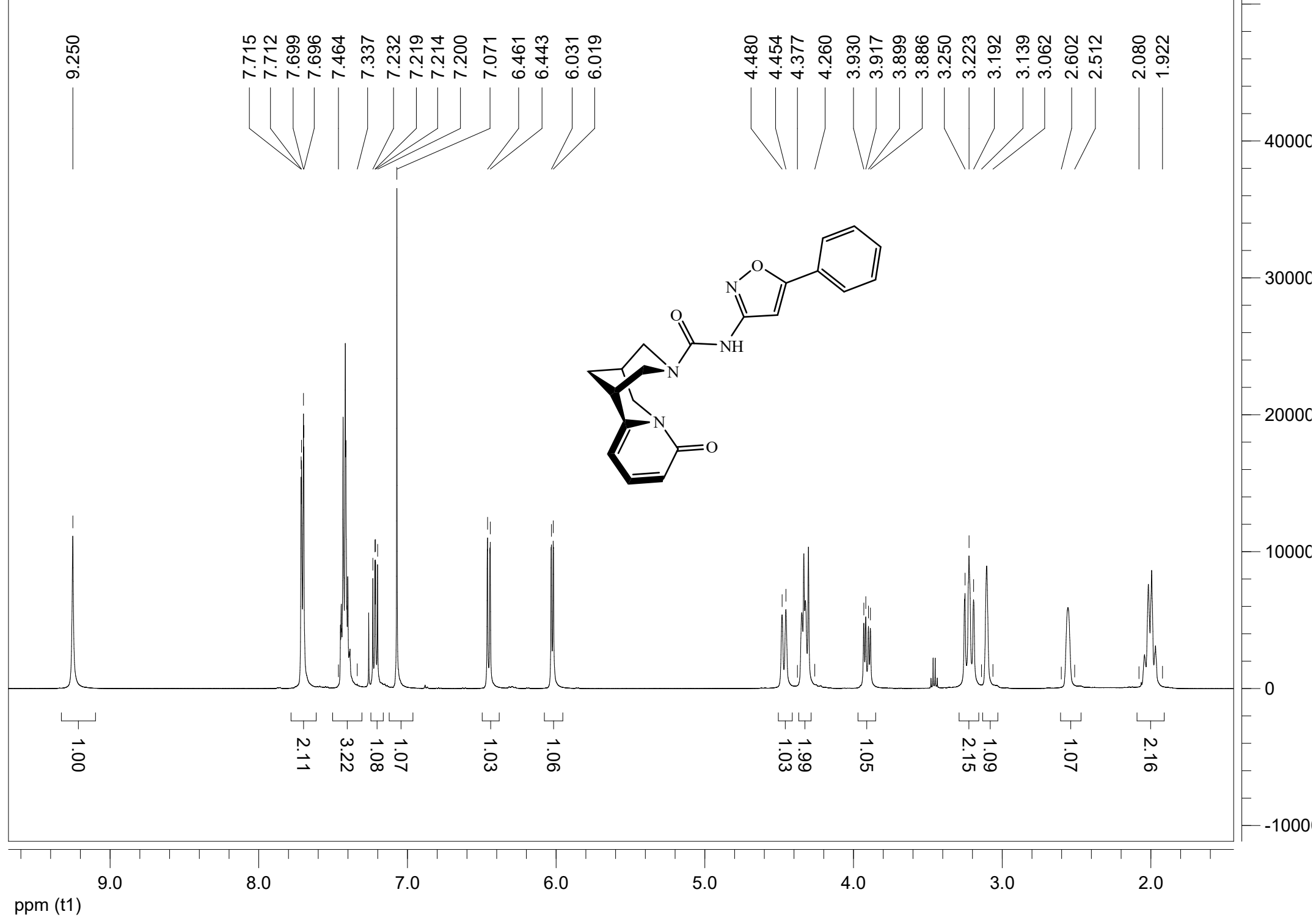

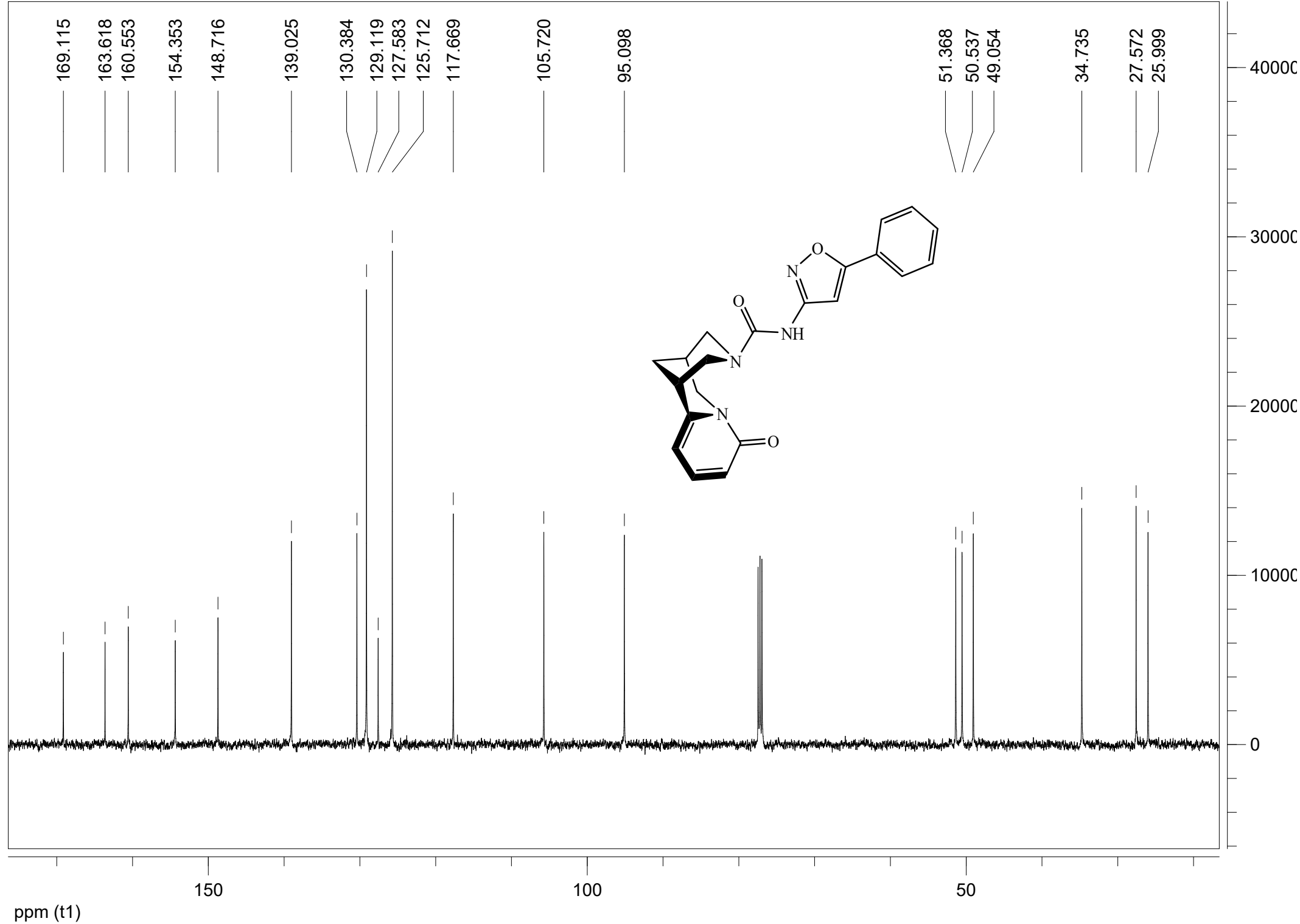

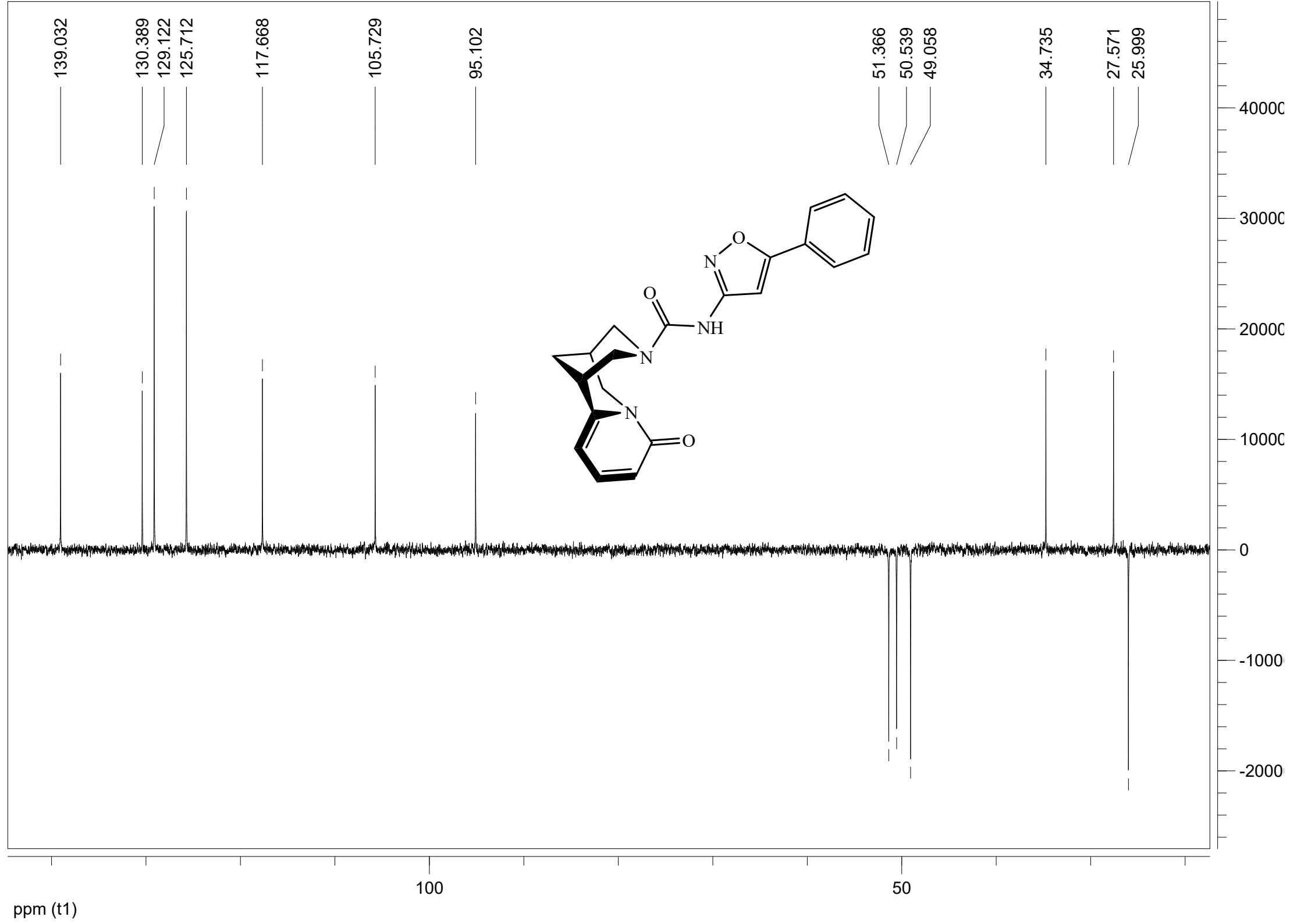

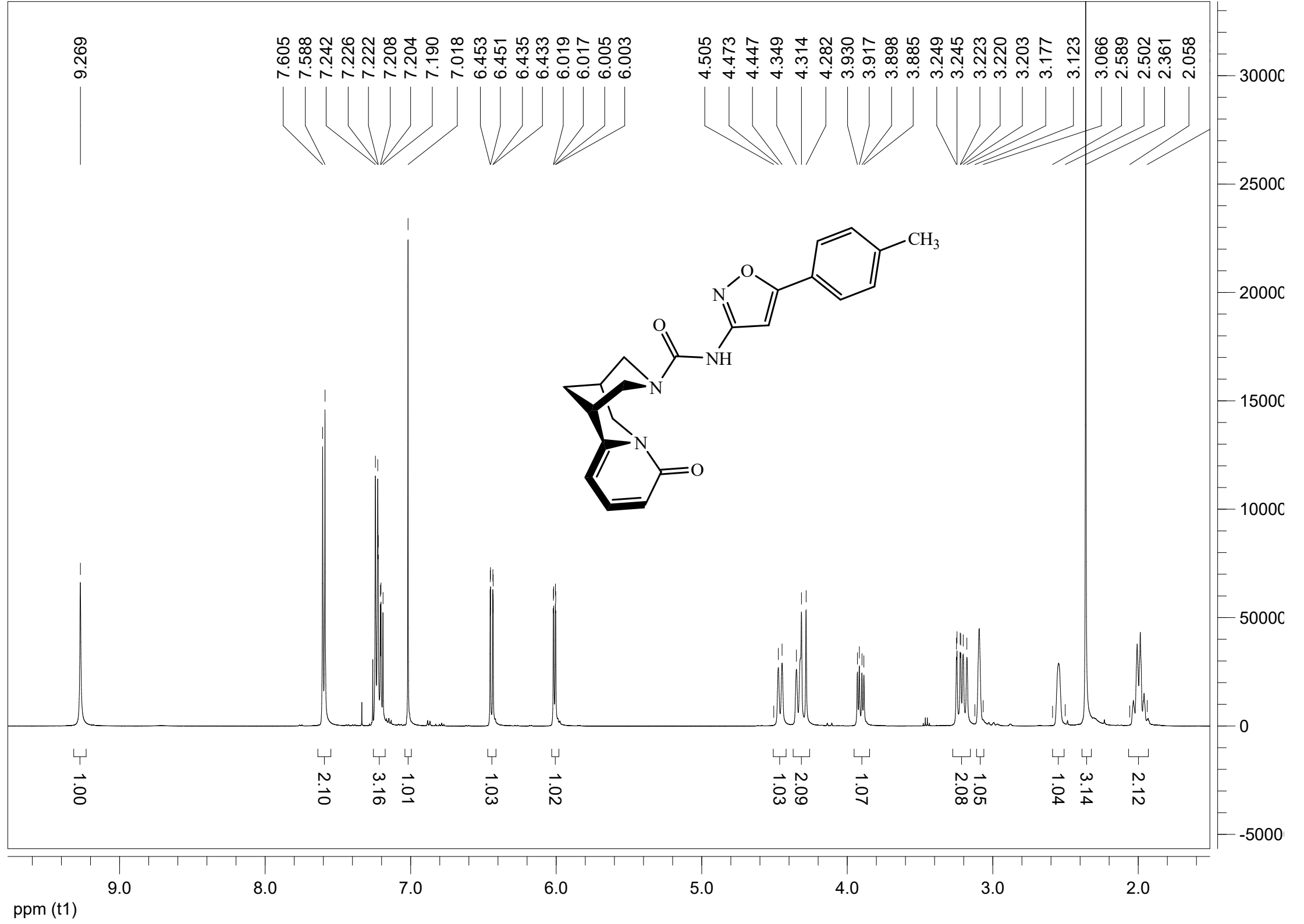

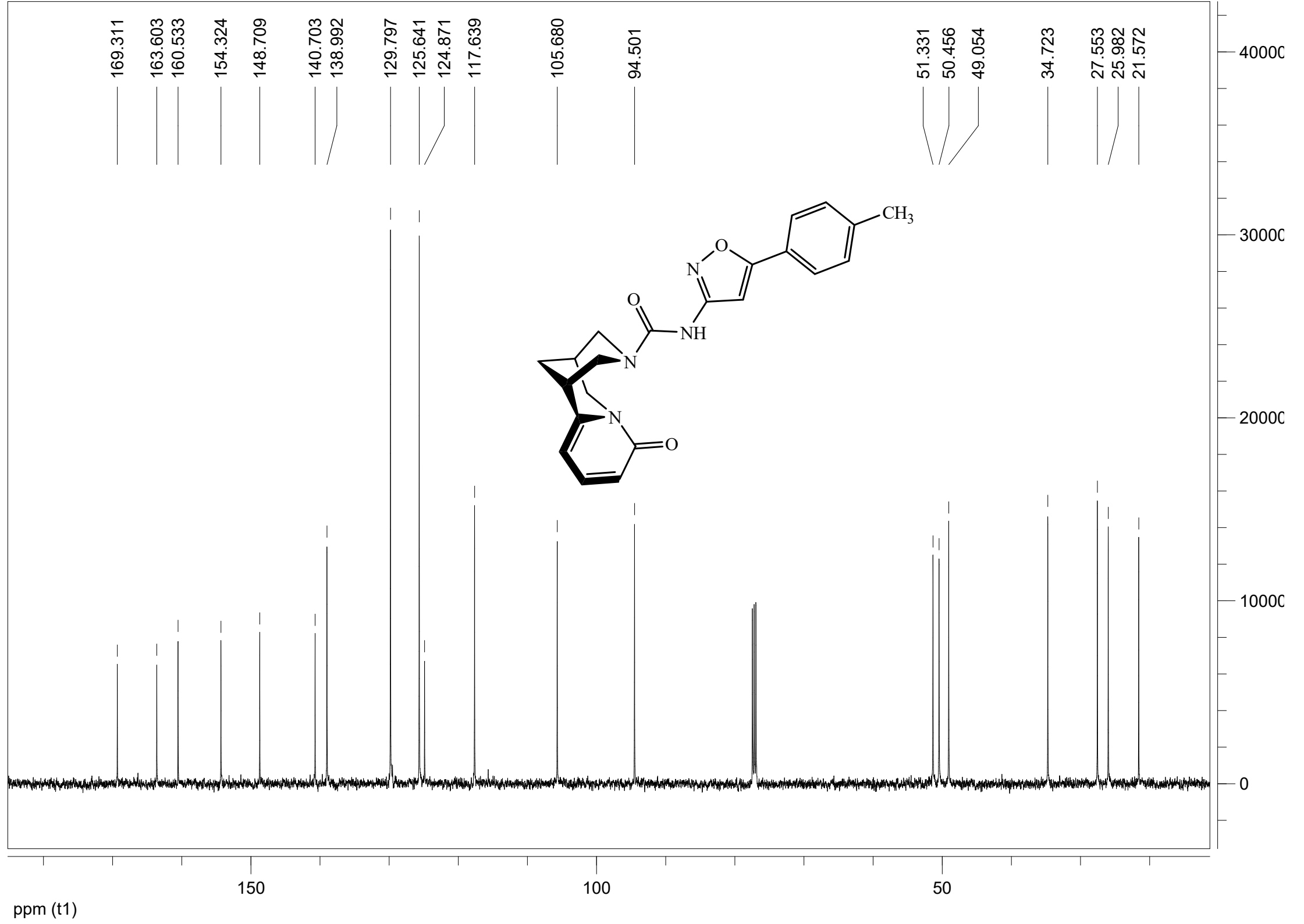

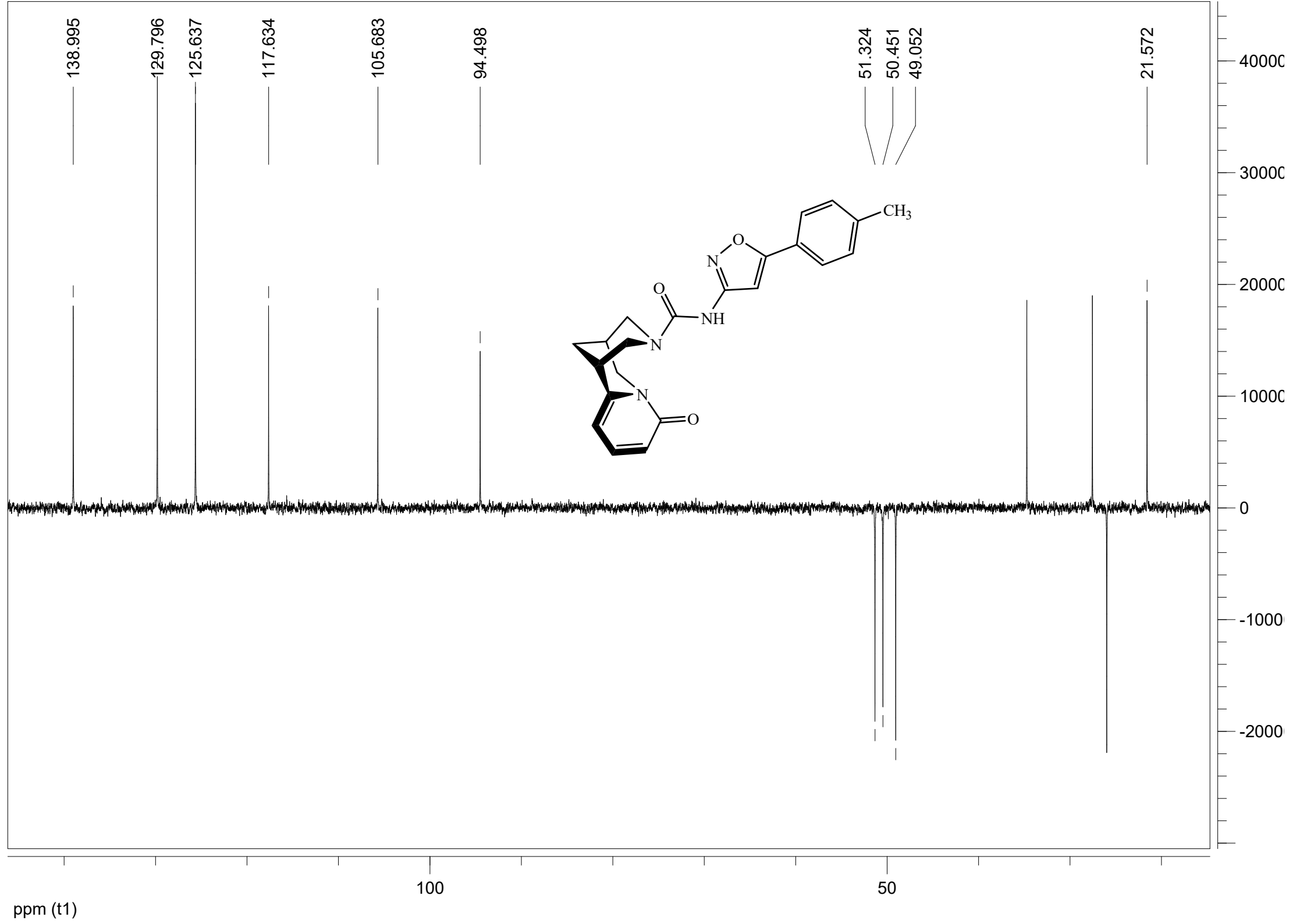

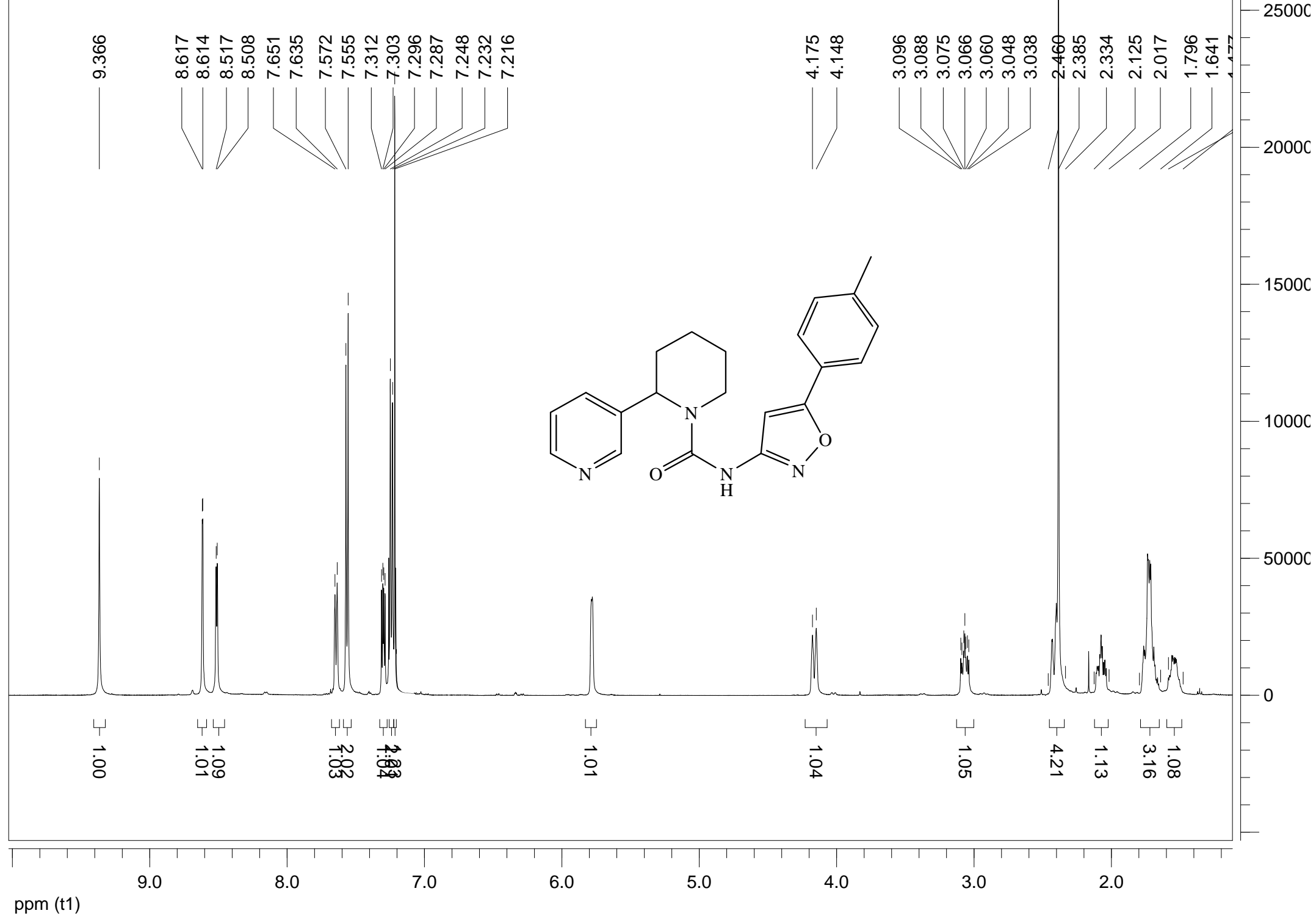

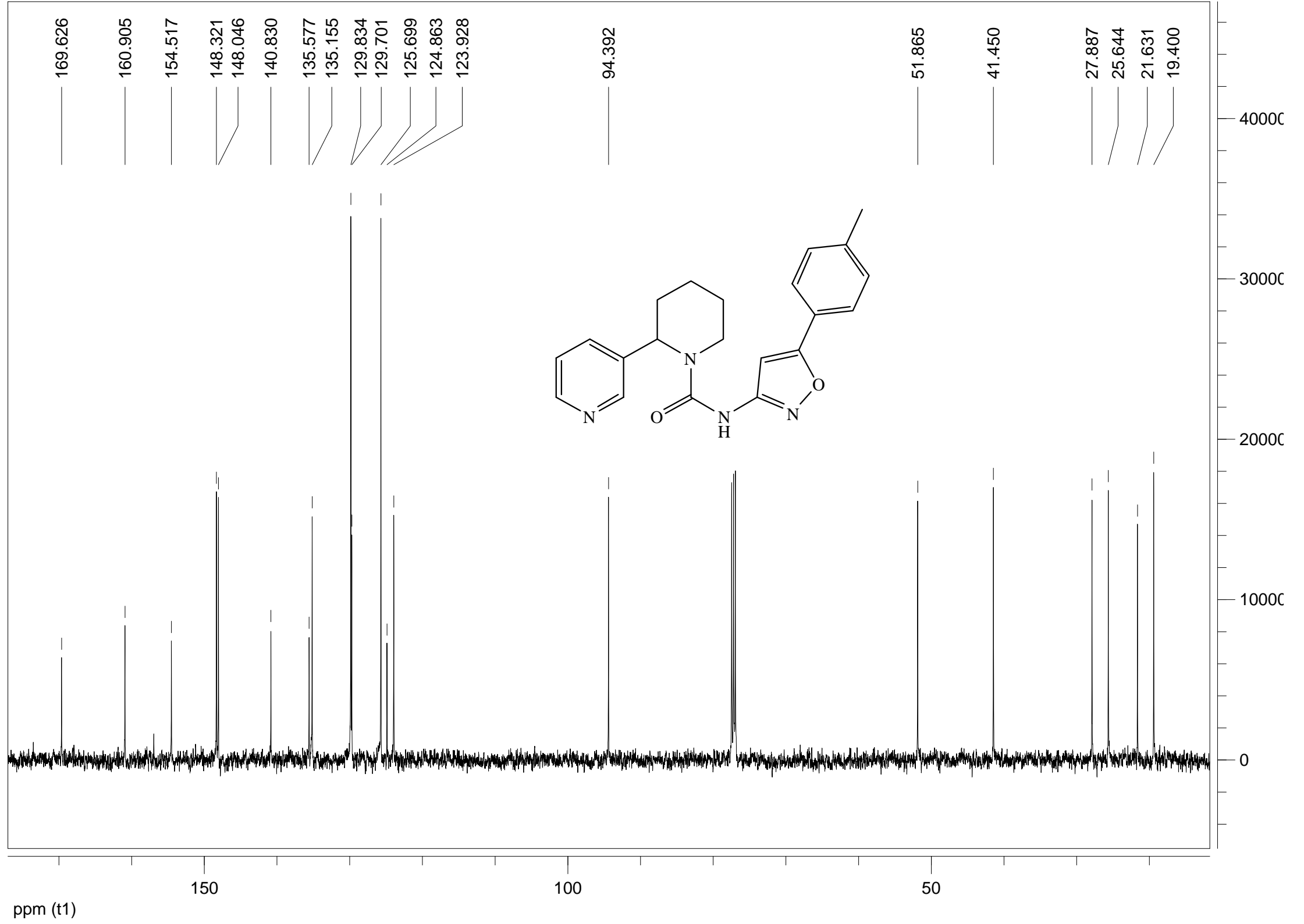

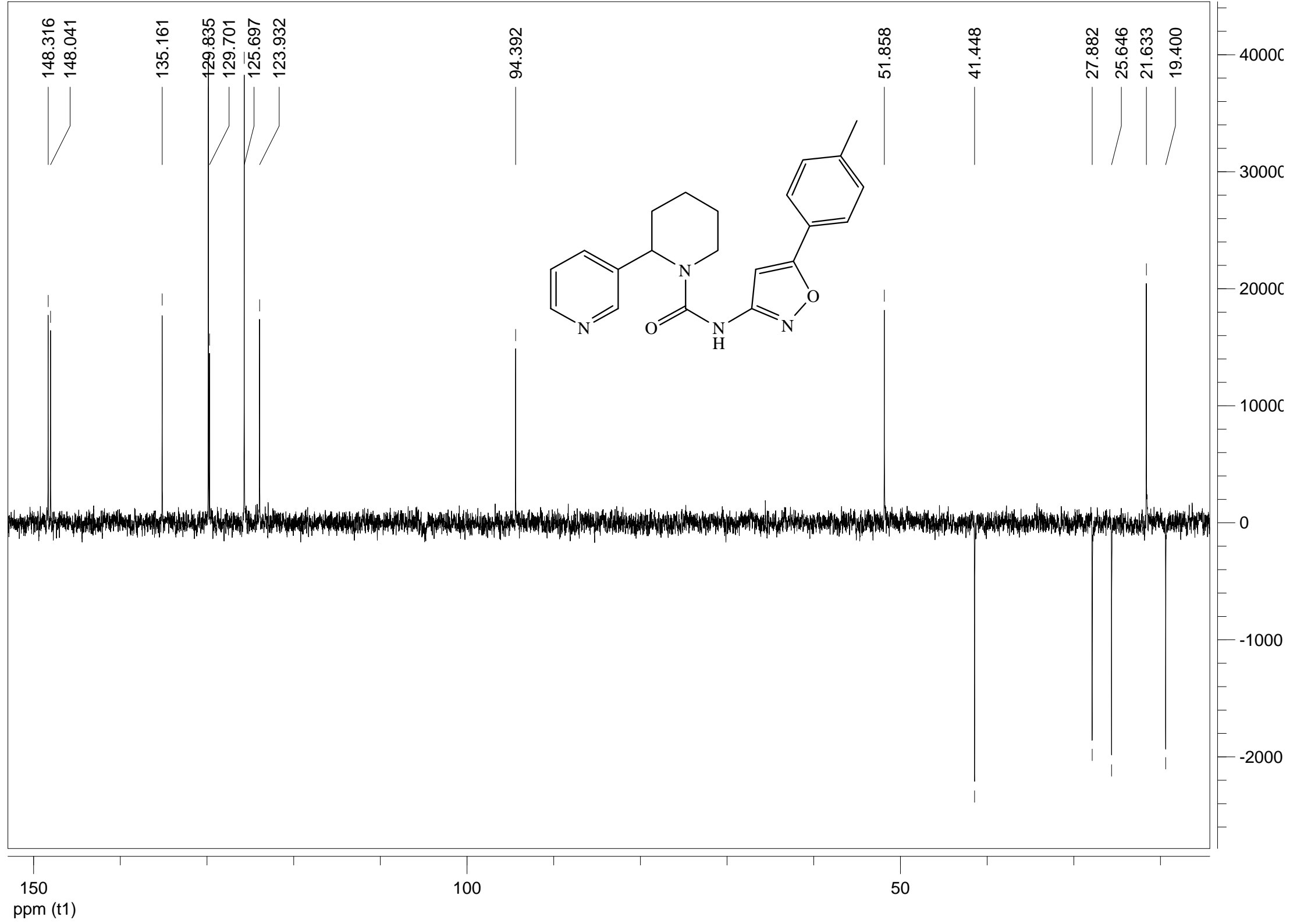

Supplement: Supplementary file 1 [file molecules-29-03246-s001.zip › NMR.pdf]
